# Targeting of intracellular oncoproteins with peptide-centric CARs

**DOI:** 10.1038/s41586-023-06706-0

**Published:** 2023-11-08

**Authors:** Mark Yarmarkovich, Quinlen F. Marshall, John M. Warrington, Rasika Premaratne, Alvin Farrel, David Groff, Wei Li, Moreno di Marco, Erin Runbeck, Hau Truong, Jugmohit S. Toor, Sarvind Tripathi, Son Nguyen, Helena Shen, Tiffany Noel, Nicole L. Church, Amber Weiner, Nathan Kendsersky, Dan Martinez, Rebecca Weisberg, Molly Christie, Laurence Eisenlohr, Kristopher R. Bosse, Dimiter S. Dimitrov, Stefan Stevanovic, Nikolaos G. Sgourakis, Ben R. Kiefel, John M. Maris

**Affiliations:** 1 Perlmutter Cancer Center, New York University Grossman School of Medicine, New York, NY USA; 2 Division of Oncology and Center for Childhood Cancer Research, Children’s Hospital of Philadelphia, Philadelphia, PA USA; 3 Myrio Tx, Melbourne, Victoria Australia; 4 Department of Biomedical and Health Informatics, Children’s Hospital of Philadelphia, Philadelphia, PA USA; 5 University of Pittsburgh, Pittsburgh, PA USA; 6 University of Tubingen, Tubingen, Germany; 7 Perelman School of Medicine at the University of Pennsylvania, Philadelphia, PA USA; 8 Department of Pathology and Lab Medicine, Children’s Hospital of Philadelphia, Philadelphia, PA USA; 9 Department of Chemistry and Biochemistry, University of California Santa Cruz, Santa Cruz, CA USA; 10 Massachusetts Institute of Technology, Cambridge, MA USA

**Keywords:** MHC class I, Cancer immunotherapy

## Abstract

The majority of oncogenic drivers are intracellular proteins, constraining their immunotherapeutic targeting to mutated peptides (neoantigens) presented by individual human leukocyte antigen (HLA) allotypes^1^. However, most cancers have a modest mutational burden that is insufficient for generating responses using neoantigen-based therapies^2,3^. Neuroblastoma is a paediatric cancer that harbours few mutations and is instead driven by epigenetically deregulated transcriptional networks^4^. Here we show that the neuroblastoma immunopeptidome is enriched with peptides derived from proteins essential for tumorigenesis. We focused on targeting the unmutated peptide QYNPIRTTF discovered on HLA-A*24:02, which is derived from the neuroblastoma-dependency gene and master transcriptional regulator *PHOX2B*. To target QYNPIRTTF, we developed peptide-centric chimeric antigen receptors (PC-CARs) through a counter panning strategy using predicted potentially cross-reactive peptides. We further proposed that PC-CARs can recognize peptides on additional HLA allotypes when presenting a similar overall molecular surface. Informed by our computational modelling results, we show that PHOX2B PC-CARs also recognize QYNPIRTTF presented by HLA-A*23:01, the most common non-A2 allele in people with African ancestry. Finally, we demonstrate potent and specific killing of neuroblastoma cells expressing these HLAs in vitro and complete tumour regression in mice. These data suggest that PC-CARs have the potential to expand the pool of immunotherapeutic targets to include non-immunogenic intracellular oncoproteins and allow targeting through additional HLA allotypes in a clinical setting.

## Abstract (web summary)

Peptide-centric chimeric antigen receptors (PC-CARs) provide a platform to address the challenges involved in targeting intracellular oncoproteins, and PC-CARs based on the neuroblastoma-dependency gene *PHOX2B* induce elimination of aggressive tumors.

## Main

The curative potential of chimeric antigen receptor (CAR) T cell-based cancer immunotherapies has been established in leukaemias, but such application in solid tumours has been limited by the paucity of known tumour-specific membrane proteins^5^. Although membrane proteins represent up to one quarter of the proteome, only a fraction of these are specifically expressed on tumour cells and not on normal tissues, and a smaller proportion are essential for tumour homeostasis^6^. Rather, most cancer-driver proteins reside in the cytoplasm or nucleus of the cell where they are accessible to the immune system only through the presentation of peptides on the major histocompatibility complex (MHC).

MHC class I proteins, encoded by the highly polymorphic human leukocyte antigen (HLA) A, B and C genes, present a snapshot of the intracellular proteome on the cell surface (immunopeptidome) where T cells surveil peptide–MHC (pMHC) complexes for antigens derived from foreign pathogens. T cell recognition of mutation-derived pMHC neoantigens as non-self is the basis of curative responses achieved through immune checkpoint blockade and complete remissions using adoptive transfer of tumour infiltrating lymphocytes^7^. Nonetheless, only about 5% of these neoantigens are predicted to bind a given HLA allotype^8^, and only 1.6% of neoantigens are reported to be immunogenic^3^. Subclonal mutations and downregulation of mutated non-essential genes further constrain the pool of therapeutically relevant neoantigens. Consequently, a mutational threshold for effective neoantigen-based therapies is necessary, which is not surpassed in most cancers^9^. Tumour cells also present a plethora of unmutated self-peptides on MHC^10^, but these are largely immunogenically silent owing to negative thymic selection of T cells. We proposed that a subset of the immunopeptidome consists of tumour-specific peptides derived from essential oncoproteins and that these can be targeted using synthetic PC-CARs.

Peptides presented in the MHC groove make up only a small fraction of the extracellular pMHC molecular surface. The typical 8–14 amino acid peptide presented on MHC class I is composed of only about 2–3% of the amino acids in the pMHC complex and is spatially confined within the adjacent α-helices of the MHC groove. This poses major challenges for engineering peptide-specific single-chain antibody variable fragment (scFv) binders^11^. Furthermore, cross-reactivity of engineered receptors with peptides of biophysically similar molecular surfaces presented in normal tissues have resulted in significant toxicity and death^12,13^.

Neuroblastoma is a childhood cancer derived from tissue of the developing sympathetic nervous system and is often lethal despite intensive cytotoxic therapy^14^. These tumours are low in mutational burden^15^ and MHC expression^16^, which makes neuroblastoma both a challenging tumour to target with MHC-based immunotherapies and an ideal model for addressing the major problems currently hindering the wider advancement of cancer immunotherapies. As a tumour-derived from neural crest progenitor cells, neuroblastomas express a set of core regulatory circuit (CRC) transcription factors involved in maintaining cell fate, metabolism, migration, epigenetic states, growth and proliferation^4^. These genes are epigenetically silenced after terminal differentiation of normal neural tissues, but these developmental pathways are aberrantly hyperactivated in neuroblastoma. Here we present the discovery of recurring lineage-restricted oncoproteins presented on MHC, focusing on immunotherapeutic targeting of the neuroblastoma CRC master regulator PHOX2B using PC-CARs.

## Identification of tumour-specific antigens

**Fig. 1:**
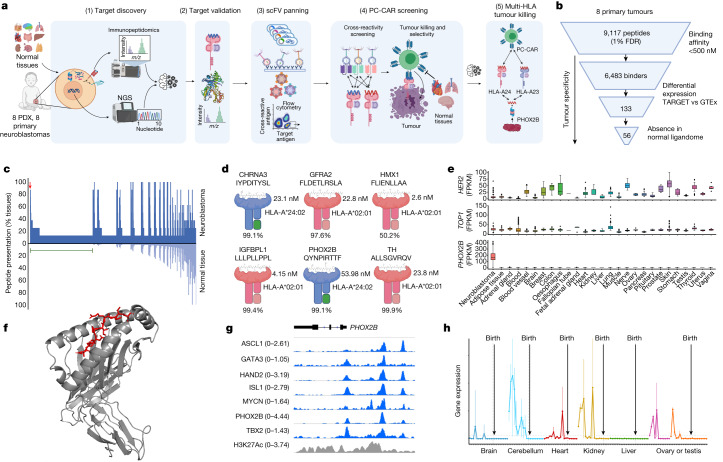
Antigen discovery and prioritization process identifies PHOX2B as an immunotherapy target. **a**, Summary of tumour antigen discovery and CAR engineering workflow: (1) integrated genomics and immunopeptidomics process, (2) target validation, (3) scFv screening, (4) CAR engineering and (5) tumour killing across HLA allotypes. NGS, next-generation sequencing. **b**, Computational filtering of 9,117 peptide instances identified by immunopeptidomics in primary tumours (1% FDR) resulted in 56 neuroblastoma-specific peptides (33 unique peptides) derived from 29 unique proteins. **c**, Primary neuroblastoma tumour immunopeptidome compared with 190 normal tissues. Each point on the *x* axis represents one of 5,832 unique peptides identified in primary tumours, with the proportion of neuroblastoma tumours presenting a given peptide annotated above the axis in dark blue and the proportion of normal tissue expressing the identical peptide below the axis in light blue. Green line overlayed with 1,492 peptides not previously observed in normal tissue immunopeptidome. Parent genes from neuroblastoma-specific peptides resulted in the top two gene ontology enrichment terms noradrenergic neuron differentiation and sympathetic nervous system development. Arrow denotes 351 recurring peptides presented in neuroblastoma not previously detected in normal tissues. **d**, Five antigens further prioritized from PDX and primary tumours by differential expression, HLA allele frequency, relative peptide abundance (percentile rank annotated below pMHC), predicted pMHC binding affinity and relevance to neuroblastoma tumorigenesis. **e**, *PHOX2B* expression in RNA sequencing of 153 neuroblastoma tumours versus 1,641 normal tissues in GTEx. *PHOX2B* expression is tumour-restricted, in contrast to the immunotherapy target *HER2* and neuroblastoma chemotherapy target *TOP1* (note differences in the *y* axis scale). Lower and upper bounds of box plots correspond to the first and third quartiles (the 25th and 75th percentiles); whiskers represent minima and maxima or 1.5× interquartile range (IQR). FPKM, fragments per kilobase million. **f**, Crystal structure of PHOX2B 9-amino-acid-long QYNPIRTTF (red) refolded with HLA-A*24:02 (grey). **g**, ChIP–seq data in neuroblastoma shows binding of all CRC proteins at the *PHOX2B* locus and association with a H3K27ac super-enhancer mark. **h**, RNA sequencing of fetal tissue demonstrates expression of PHOX2B in early development and downregulated before birth across seven tissues. Panels **a** and **d** were created using BioRender (https://biorender.com).

First, we surveyed the landscape of peptides accessible to T cells by performing MHC capture, peptide elution and liquid chromatography with tandem mass spectrometry^17^ (LC–MS/MS; immunopeptidomics) on eight neuroblastoma cell-derived xenografts (CDX) and patient-derived xenografts (PDX). These xenografts have a wide range of MHC expression and encompass the array of rare recurring mutations found in high-risk neuroblastoma^18,19^ (Fig. 1a and Extended Data Table 1a). We identified a total of 7,608 peptides from 8 tumours (1% false discovery rate (FDR); Supplementary Table 1). We did not find any of the 4,105 potential 8–14-amino-acid-long neoantigens imputed from tumour mutational data in the immunopeptidome, consistent with expected rates of neoantigen presentation and limited detection using ligandomics in low mutational tumours^20^. We first filtered the 7,608 peptides to select HLA binders with sufficient affinity to act as T cell epitopes using a predicted pMHC complex IC_50_ threshold of 500 nM, which produced 2,286 predicted strong binders. We then filtered for peptides derived from differentially expressed parent genes as determined from RNA sequencing data from 153 neuroblastoma tumours compared with 1,641 normal tissues (from the TARGET^21^ and GTEx^17^ databases, respectively). This analysis resulted in 61 peptides derived from genes with mRNA expression levels that were one log fold greater in the tumour for each normal tissue (*P* < 0.01). Finally, we filtered the remaining tumour peptides against a database of MHC peptides empirically characterized on 190 normal tissues^22^, removing any peptide with a parent gene represented in the normal tissue immunopeptidome. Although this last step does not absolutely exclude potential presentation of a peptide in normal tissues, it enabled us to narrow our list to 13 peptides derived from 9 genes expressed in neuroblastoma that have not been previously detected in any normal tissue.

**Extended Data Fig. 1:**
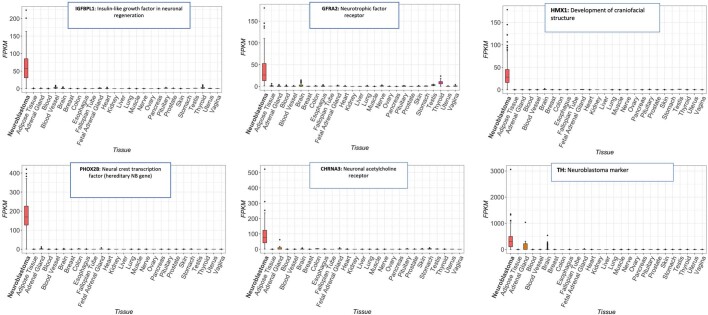
Prioritized peptides from immunopeptidome are highly differentially expressed in neuroblastoma. Differential expression between 153 neuroblastoma tumors from TARGET compared to 1643 normal tissues in GTEx, as described in Fig. 1e. Lower and upper bounds of boxplot correspond to the first and third quartiles (the 25th and 75th percentiles); whiskers represent minima/maxima or 1.5*IQR.

We then performed immunopeptidomics on eight high-risk diagnostic neuroblastoma primary tumours, focusing on HLA-A*02:01 and HLA-A*24:02 allotypes (Extended Data Table 1 and Supplementary Table 1). Using the same filtering steps as described for xenograft tumours, we identified 56 peptides (33 unique peptides derived from 29 unique proteins), with strong HLA binding affinity derived from differentially expressed genes in neuroblastoma and not previously detected in the benign tissue ligandome (Fig. 1b and Extended Data Fig. 1). We confirmed the presence of 7 out of the 13 peptides from the xenografts in primary tumours, which suggested that PDX and CDX tumours can be used as predictive models for primary tumour ligandomes. Notably, the most enriched gene ontologies of the peptide parent genes not previously observed in the normal tissues ligandomic dataset were noradrenergic neuron differentiation (*P* = 3.42 × 10^−4^, FDR = 0.0389) and sympathetic nervous system development (*P* = 2.95 × 10^−5^, FDR = 0.00665; Fig. 1c, green bar). These findings highlight the distinctiveness of the neuroblastoma ligandome and suggest that MHC-presented peptides are enriched for those derived from aberrantly expressed lineage-restricted genes.

**Extended Data Fig. 2:**
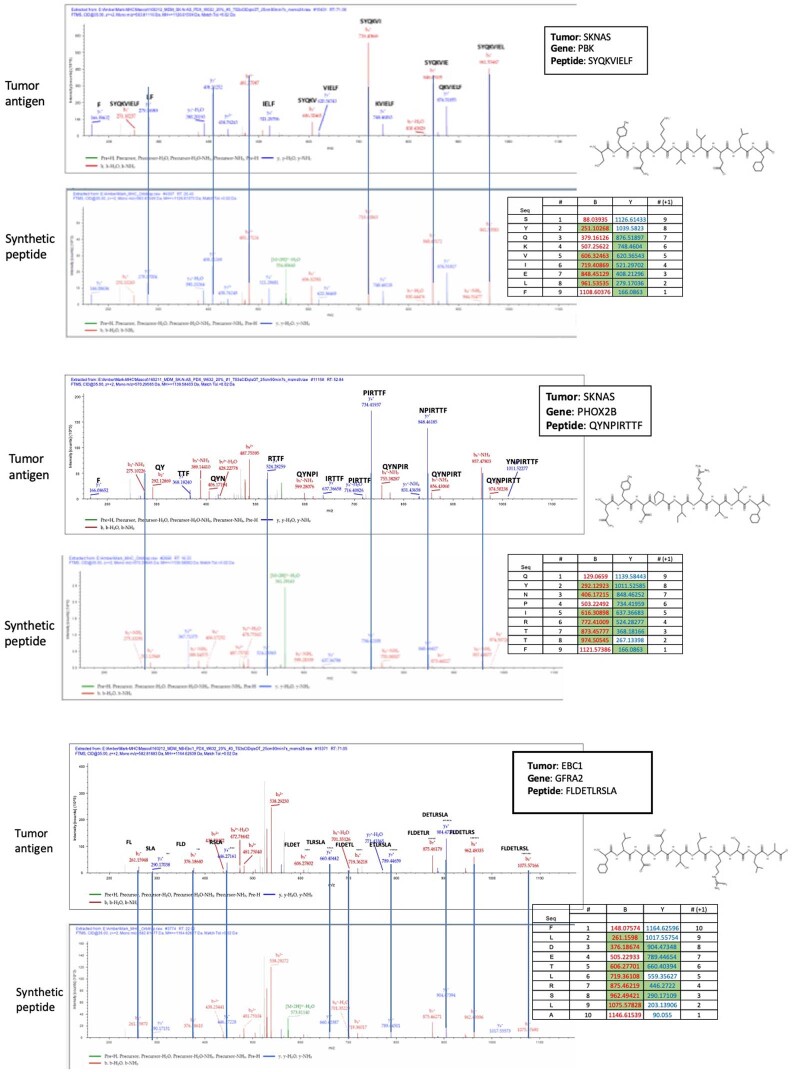
Validation of antigens discovered by immunopeptidomics using LC/MS/MS of synthetic peptide. Peptide sequences imputed from immunopeptidomics spectra were synthesized and LC/MS/MS was under matched conditions. Synthetic peptides show complete concordance with tumor-eluted peptides across all b and y ions; masses highlighted in green represent detected peaks corresponding to matched b and y ions found in both tumor-eluted peptide spectra and synthetic peptide spectra.

**Extended Data Fig. 3:**
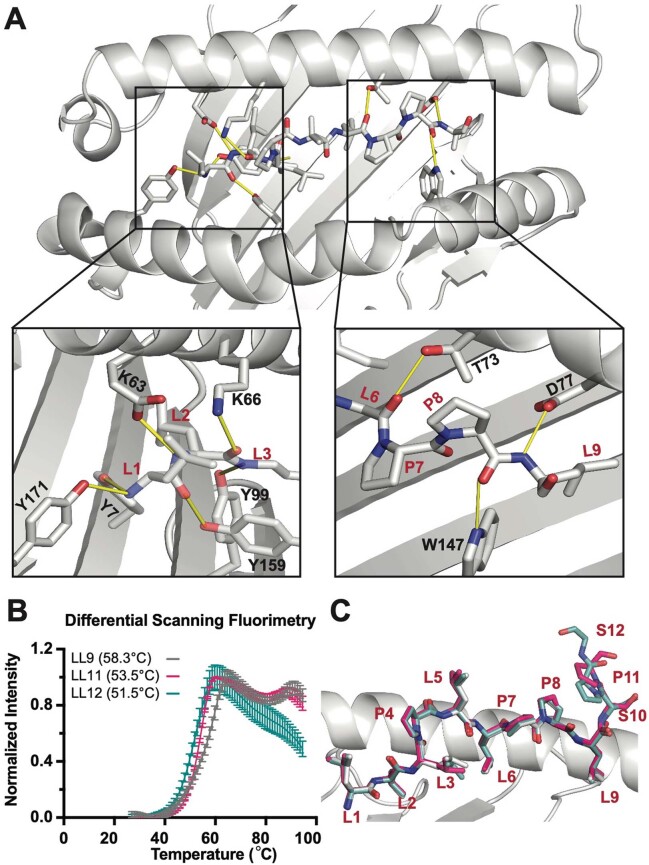
Crystal structures solved for IGFBPL1 presented as three distinct peptides on HLA-A2. Crystallographic analysis of IGFBPL1 peptide bound to HLA-A*02:01. a. X-ray structure of HLA-A*02:01 presenting the IGFBPL1 nonameric peptide (LLLPLLPPL), where yellow lines represent polar contacts between the HLA groove and peptide. b. Differential Scanning Fluorimetry (DSF) of HLA-A*02:01 refolded with IGFBPL1 peptides of different lengths. The legend indicates the sequence of the IGFBPL1 peptide and the corresponding melting temperature of the resulting peptide/MHC-I complexes. Mean of triplicate samples reported with error bars representing SD. c. Overlay of IGFBPL1 9mer, 11mer, and 12mer in MHC groove reveals that core peptides and anchor residues are maintained across peptides of varying length, and that additional amino acids in the 11mer and 12mer protrude at C terminus downstream of the L9 anchor position.

To select peptides with the highest potential as putative immunotherapeutic targets, we prioritized peptides on the basis of pMHC complex binding affinity, HLA allele frequency, degree of differential expression of parent genes, relative abundance on MHC compared with other peptides, recurrence across multiple neuroblastoma tumours and relevance to neuroblastoma biology based on the published literature^23–25^. One peptide each from CHRNA3, GFRA2, HMX1, IGFBPL1, PHOX2B and TH was selected for preclinical development (Fig. 1d,e and Supplementary Table 1). The presence of these peptides in tumours was validated by performing LC–MS/MS on synthetic peptides (Extended Data Fig. 2), which produced complete concordance across *b* and *y* ions. In addition, we validated peptide binding to predicted HLA alleles by refolding the pMHC complex and solving the crystal structures of one PHOX2B peptide–HLA-A*24:02 (Protein Data Bank (PDB) identifier: 7MJA) and three IGFBPL1 peptide–HLA-A*02:01 (PDB identifiers 7MJ6, 7MJ67 and 7MJ8) protein complexes (Fig. 1f and Extended Data Fig. 3b). Of note, we detected three distinct IGFBPL1 peptides as 9, 11 and 12 amino acids long (all with the same core 9 amino acids) as stable pMHC class I complexes (Extended Data Fig. 3). This result provides support for a previous study^26^ demonstrating that multiple peptides with distinct conformations may arise from the same region of a protein and present multiple opportunities for therapeutic targeting.

**Extended Data Fig. 4:**
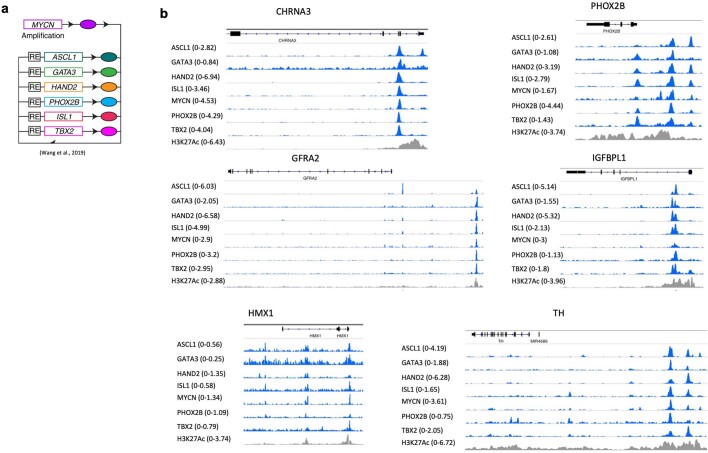
Tumor antigens derived from parent genes are under control of neuroblastoma core regulatory circuit. **a**. Illustration of core-regulatory circuit in which transcription factors bind to one another’s promoters, acting as feed-forward loop **b**. ChIP Seq data at prioritized neuroblastoma antigens parent gene loci are bound by each of the 7 core-regulatory circuit (CRC) proteins *MYCN, ASCL1, GATA3, HAND2, PHOX2B, ISL1, and TBX2*. All CRC binding sites are associated with a H3K27Ac super-enhancer element.

**Extended Data Fig. 5:**
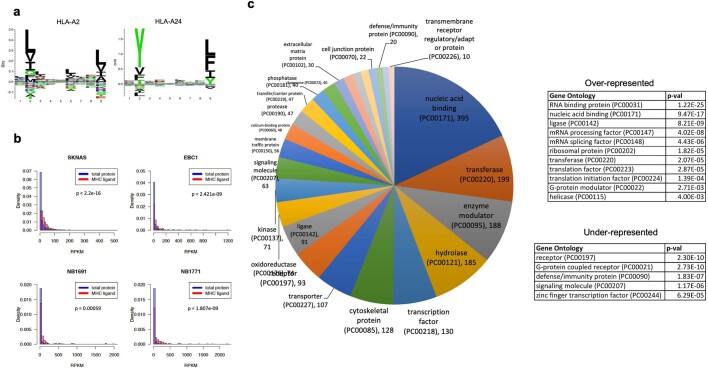
Properties of neuroblastoma immunopeptidome. **a**. Logo plots of peptides eluted from HLA-A2 and HLA-A24 show canonical binding motifs for both alleles^64^. **b**. Peptides detected by immunopeptidomics are enriched for highly expressed parent genes as compared to the entire cellular transcriptome (p = 2.2 × 10^−16^ – 0.00059 across cell lines). **c**. Gene ontology analysis of the parent genes of peptides of the neuroblastoma immunopeptidome compared to the cellular transcriptome. The most significantly enriched ontology groups are nucleic acid binding proteins (RNA binding protein ontology p = 1.22e^−25^ and nucleic acid binding ontology p = 9.47e^−17^; Gene Ontology analyses were performed using PANTHER^41^ (http://geneontology.org/) and p-values calculated using Fischer’s Exact test).

**Extended Data Fig. 6:**
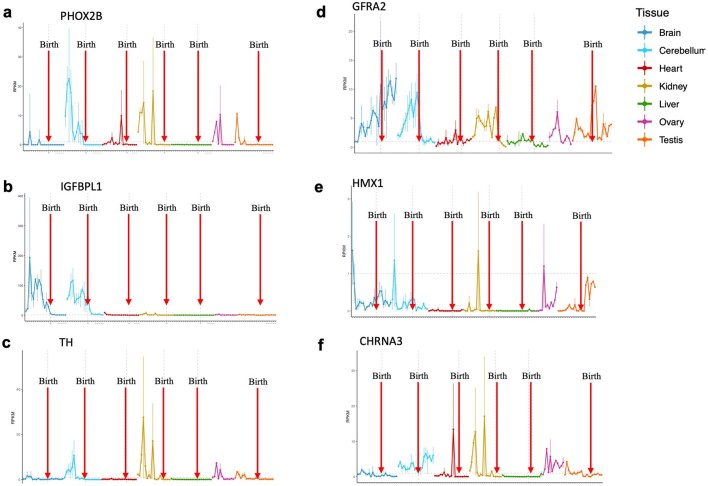
Prioritized antigen parent genes are expressed during tissue development and downregulated in normal tissue after birth. **a**-**f**. Temporal transcriptomic analysis of prioritized neuroblastoma target antigens reveals high expression during development and downregulation prior to birth in the majority of target genes. Expression shown for each tissue from shown from 4 weeks post-conception to 58–63 years with birth marked by red arrows. PHOX2B (a), TH (b), IGFBPL1 (c), and CHRNA3 (d) exhibit developmentally restricted expression patterns. HMX1 (e) shows expression in testes post-birth. GFRA2 (f) expression in brain may make this target more amenable to targeting by antibody-based therapies that do not cross blood-brain barrier.

We then inferred the ability of a tumour to evade the immune response through the downregulation of target genes by examining the binding of neuroblastoma CRC transcription factors (MYCN, ASCL1, HAND2, ISL1, PHOX2B, GATA3 and TBX2; Extended Data Fig. 4a) to the parent gene locus of the prioritized antigens^4^. All six CRC proteins bound regulatory elements at each parent gene loci within H3K27ac super-enhancer elements (Fig. 1g and Extended Data Fig. 4b), which suggested that transcriptional redundancy and dependency should mitigate the risk of antigen loss in response to immunotherapy due to downregulation of the parent gene. In addition, peptides from each of the six CRC proteins were represented in the neuroblastoma immunopeptidome (Extended Data Table 1b). Indeed, the most significantly enriched gene groups in the immunopeptidome were nucleic acid-binding proteins (Extended Data Fig. 5c; *P* = 9.5 × 10^−17^). Finally, we interrogated the dynamics of gene expression during development using temporal transcriptomics data^27^. Consistent with its function in orchestrating neural crest progenitor development^28^, PHOX2B was expressed exclusively during fetal development and was completely silenced in normal tissues before birth (Fig. 1h), as were IGFBPL1, TH and CHRNA3 (Extended Data Fig. 6). PHOX2B is a key CRC protein that is among the most specifically expressed genes in neuroblastoma^29^. PHOX2B expression was not detected in normal tissue, in constrast with many solid tumour immunotherapy targets, including HER2, or chemotherapy (such as camptothecin) targets in neuroblastoma, including TOP1, each of which demonstrates significant expression in normal tissue (Fig. 1e). PHOX2B expression is routinely used in diagnostic assays for neuroblastoma^30^, is one of two highly penetrant susceptibility genes in this tumour^31^ and is the third most significant dependency in neuroblastoma as reported in DepMap^32^. Taken together, we suggest that PHOX2B is a highly specific tumour antigen in neuroblastoma and an ideal candidate for therapeutic targeting.

**Extended Data Fig. 7:**
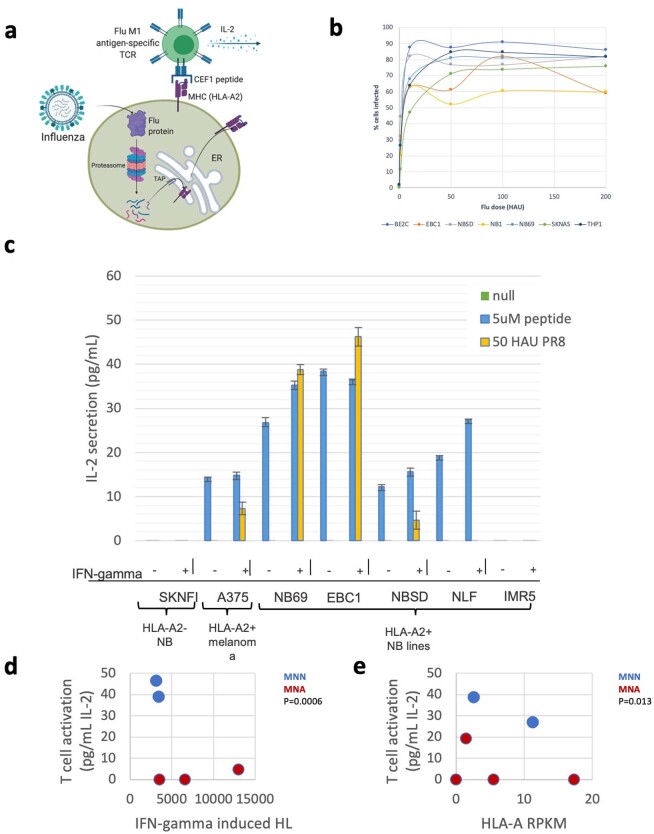
Neuroblastoma antigen processing, presentation, and immunogenicity. **a**. Schematic of immunogenicity experiment. HLA-A2 neuroblastoma cells were either infected with H1N5 or pulsed with synthetic CEF1 peptide and co-cultured with M1 antigen-specific T cell hybridoma line. T cell activation was evaluated by IL-2 release. **b**. Viral titration of neuroblastoma cells using H1N5 influenza virus measured by FACS staining of viral nucleoprotein expression at 0–200 heamagglutination (HAU). **c**. Experimental schematic of assay: 1) HLA-A2 neuroblastoma cells are pulsed with 5 μM CEF1 peptide and co-cultured with M1 antigen-specific hybridoma and IL-2 release is measured by ELISA; 2) Neuroblastoma cells are infected with 50 HAU of H1N5, co-cultured with M1 antigen-specific hybridoma, measured by IL-2 secretion. Four of seven tested HLA-A*02:01 elicit T hybridoma response when pulsed with CEF1 peptide; three of seven lines induce a response when infected with H1N5 virus. n = 3 independent experiments, each performed in triplicate; plots show mean +/− SD. **d**-**e**. T hybridoma activation is not associated with HLA expression, but activation is lower in MYCN amplified tumors (MNA) as compared to non-amplified tumors (MNN). Created with BioRender.com.

Before developing an immunotherapeutic construct that targets the PHOX2B peptide, we validated that low MHC expression in neuroblastoma^16^ did not prohibit T cell engagement and activation using an influenza antigen model (experimental details in Extended Data Fig. 7).

## PC-CAR T cell engineering for PHOX2B

**Extended Data Fig. 8:**
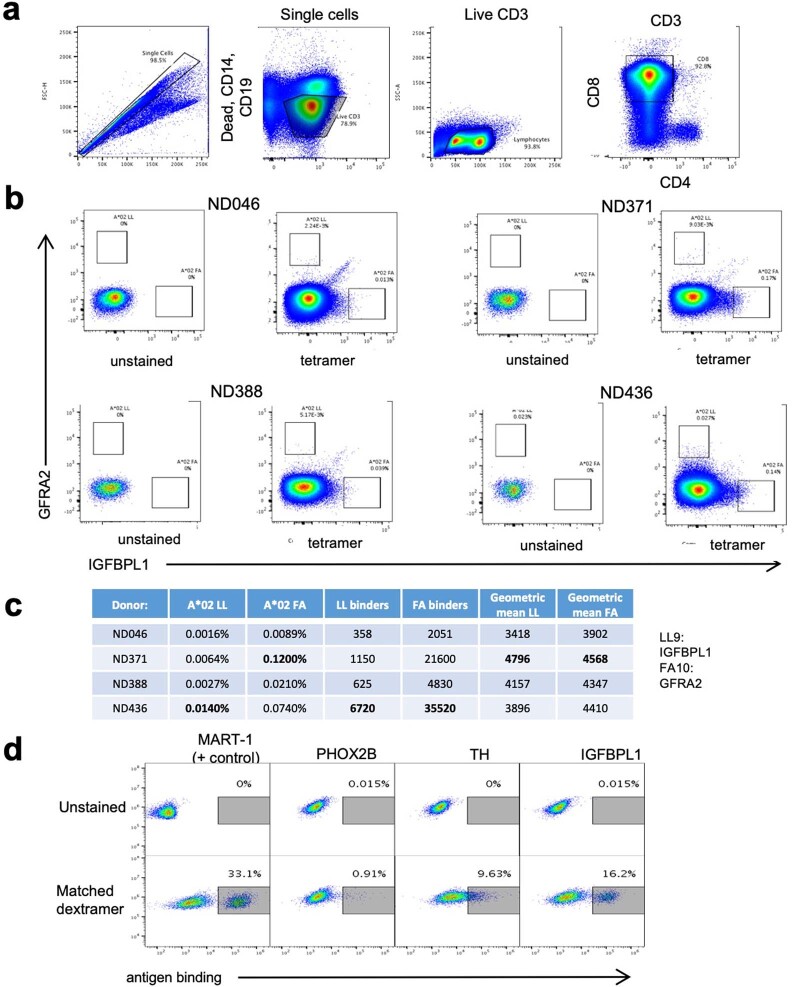
Detection of tumor self-antigen specific CD8 T cells in normal donors. **a**. Gating used to select CD8 T cells. Similar gating strategy was used to select live singlets for Jurkat and primary cells transduced with CAR constructs (without selection for CD3 and exclusion of CD14, CD19, and CD4). **b**. Four normal donors stained with IGFBPL1 dextramer on x-axis and GFRA2 on y-axis shows rare population of antigen-specific cells varying by donor and antigen (left panel showing unstained and right panel showing tetramer stained). **c**. Frequencies and MFIs of antigen specific cells across donors. **d**. Top constructs generated from normal donor-derived antigen-specific TCRs found by single-cell sequencing show range of antigen binding PHOX2B, TH, and IGFBPL1 as compared to DMF5 receptor targeting MART-1. Screening for antigen specific T cells in 3 donors reveals that PHOX2B TCRs have minimal target binding, suggesting that PHOX2B self-antigen is immunogenically silent and warrants targeting using synthetic scFv receptors.

**Extended Data Fig. 9:**
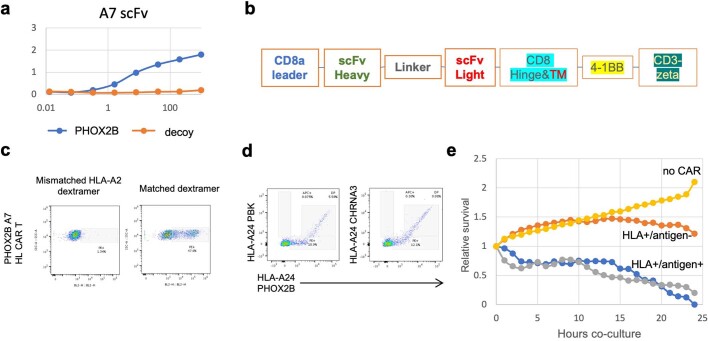
Development of antigen-specific CARs for neuroblastoma antigens. **a**. ELISA of PHOX2B scFv A7 using PHOX2B p/MHC and decoy peptide on HLA-A*24:02. **b**. Schematic of second-generation CAR constructs. **c**. A7 CAR transduced into primary CD8 cells binds PHOX2B dextramer but not HLA-A*02:01 dextramer. **d**. A7 CAR preferentially binds PHOX2B dextramer but cross-reacts with mismatched peptides PBK and CHRNA3 on HLA-A*24:02 at high affinity. **e**. A7 CAR potently kills HLA-A*24:02 neuroblastoma lines, but also kills HLA^+^/antigen^−^ tumor cells. **f**. Tetramer and dextramer gating strategy for pMHC staining.

**Extended Data Fig. 10:**
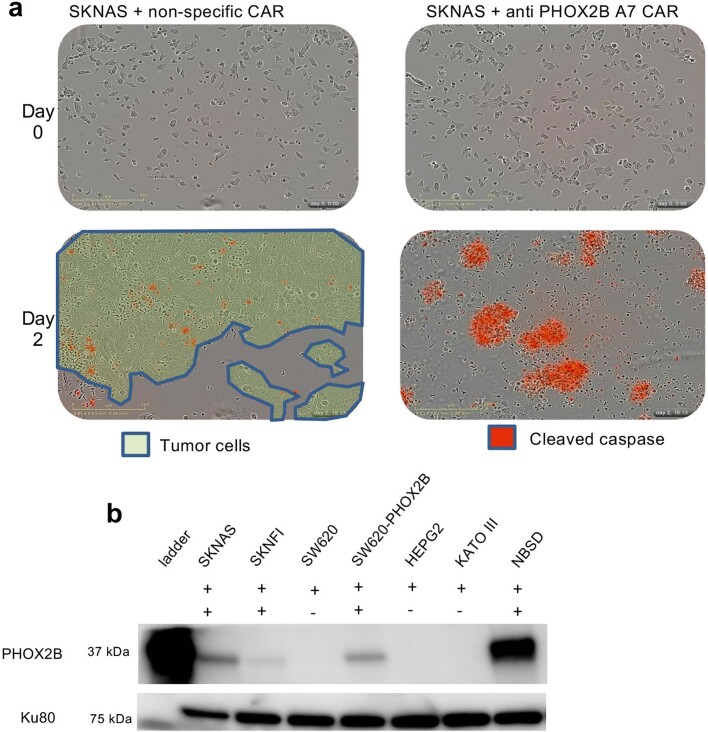
PHOX2B A7 CAR. **a**. SKNAS tumor cells co-cultured with A7 CAR shows potent killing. SKNAS cells plated on day 0 (top), non-targeting CAR (left) and A7 CAR (right) added after 18 h; measuring tumor confluence (green) and cleaved caspase (red) on day 2 (bottom) shows tumor outgrowth with non-specific CAR and killing of all tumor cells with A7 CAR. Representative images shown from experiment repeated three times using n = 3 replicates in each experiment. **b**. PHOX2B is expressed in neuroblastoma cell lines (SKNAS, SKNFI, and NBSD), and not in HLA-matched controls (SW620, KATO III, and HEPG2). PHOX2B is expressed in SW620 cells transduced with full-length PHOX2B. For gel source data from single experiment, see Supplementary Fig. 1.

**Extended Data Fig. 11:**
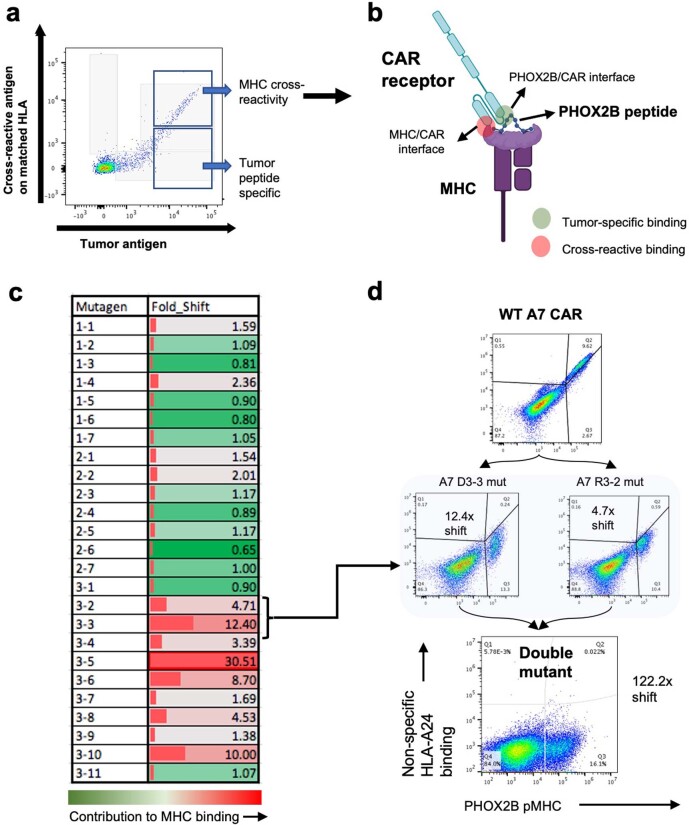
Saturation mutagenesis of A7 construct resulted in single-antigen-specific population. **a**-**b**. Cross-reactive binding to mismatched HLA-A24 peptides. Flow cytometry of Jurkat cells transduced with A7 CAR stained with PHOX2B dextramer on x-axis and mismatched PBK peptide on HLA-A*24:02 on y-axis. **c**. Saturation mutagenesis was performed on CDR loops 1–3 of the heavy chain. Each pool of mutants was stained with target pMHC and counter-stained with mismatched pMHC. Contribution of each CDR binding loop (mutagens labeled CDR loop # - position #) to binding HLA-A*24:02 is shown (green: no contribution to binding; red: significant HLA binding at specific amino acid). Contribution of each position to HLA binding was calculated as follows: (MFI_target(mut)_/MFI_mismatch(mut)_)/(MFI_target(WT)_/MFI_mismatch(WT)_). **d**. Mutations of A7 CAR at CDR3 positions 2 and 3 result in 12.4x and 4.7x shifts towards single specificity, respectively. Double mutation of D3A and R2A resulted in modified A7 CAR with no MHC cross-reactivity and reduced binding to target. Created with BioRender.com.

Owing to the lack of immunogenicity of self-antigens, we pursued the development of scFv-based CARs rather than engineered T cell receptors (TCRs) for PHOX2B after no high-affinity TCRs were identified in multiple screens (Extended Data Fig. 8). We reasoned that immunogenicity could be induced to otherwise immunogenically inert pMHC complexes using synthetic, peptide-centric receptors. Our first generation of pMHC-directed CARs demonstrated insignificant cross-reactivity to the MHC, which we were able to abrogate using saturation mutagenesis techniques (described in Extended Data Figs. 9–11).

**Fig. 2:**
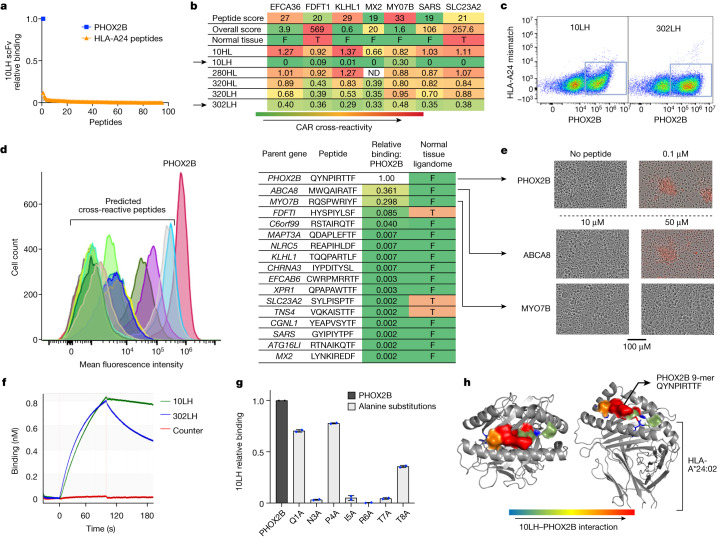
Engineering pMHC-specific CARs. **a**, Ranked binding affinity of 10LH scFv to PHOX2B and a panel of 95 peptides presented on HLA-A*24:02 peptides demonstrate high target binding and negligible binding to HLA-A*24:02 pMHC complexes. **b**, Cross-reactivity algorithm identifies CAR constructs with significant off-target binding and informs prioritization of highly selective receptors (selective receptors marked with arrows). Peptide score represents the predicted cross-reactivity based on the amino acid sequences of normal tissue peptides; overall score calculated based on peptide score, binding affinity and normal tissue expression. F, absent in normal immunopeptidome; T, peptides reported in the normal tissue immunopeptidome. **c**, Example counterstaining of top CAR clones with target (*x* axis) and off-target (*y* axis) peptides on HLA-A*24:02 reveals selective target binding in 10LH and 302LH constructs. **d**, Flow cytometry plot (left) of predicted cross-reactive peptides compared with PHOX2B shows cross-reactive binders ABCA8 (light blue) and MYO7B (gray). FDFTI is in purple and C6orf99 in dark olive green. Flow mean fluorescence intensity quantified by relative binding to PHOX2B is in table; right. **e**, Functional screening of ABCA8 and MYO7B shows CAR killing only through ABCA8 at a supraphysiological concentration of 50 µM compared with PHOX2B killing at 0.1 µM. ABCA8 and MYO7B were not detected in the normal tissue immunopeptidome, and none of the peptides predicted by sCRAP that were detected in the normal immunopeptidome (FDFTI, SLC23A2 and TNS4) demonstrate binding to 10LH. Experiment was performed once on entire panel of CAR constructs and repeated for 10LH and 302LH on expanded panel of peptides. **f**, Representative BLItz plot at 200 nM shows a fast on-rate for 10LH and 302LH and slow off-rate for 10LH (*K*_d_ = 7.6 × 10^−4^ s^–1^). **g**, Alanine scan of QYNPIRTTF reveals that mutations in five residues (N3A, I5A, R6A, T7A and T8A) result in significant abrogation of binding to PC-CAR 10LH (*n* = 2; data presented as mean). **h**, PHOX2B–HLA-A*24:02 crystal structure paired with alanine scan of 10LH using MHC class I tetramers allows mapping of the peptide–receptor interface, revealing spatial conformation of five receptor contact residues.

**Extended Data Fig. 12:**
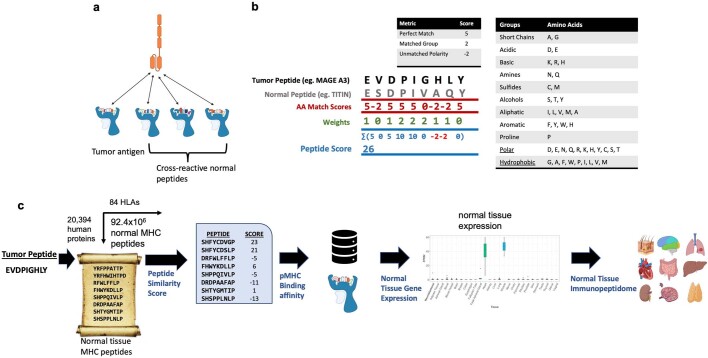
sCRAP cross-reactivity algorithm. **a**. Cross-reactivity algorithm was developed to identify peptides presented on normal tissue with similar biophysical properties to tumor antigens such as to pre-emptively predict cross-reactivities and screen for specificity. **b**. Illustration of peptide scoring system described in methods. **c**. Schematic of algorithm workflow describing how tumor peptides are scored against each peptide predicted to be presented from the normal proteome (totaling 92.4 × 10^6^ potential MHC peptides). Binding affinity is predicted for each normal peptide and maximum gene expression of parent gene are factored into the overall score of each peptide. Peptides are referenced against a normal tissue immunopeptidomics database. Created with BioRender.com.

**Extended Data Fig. 13:**
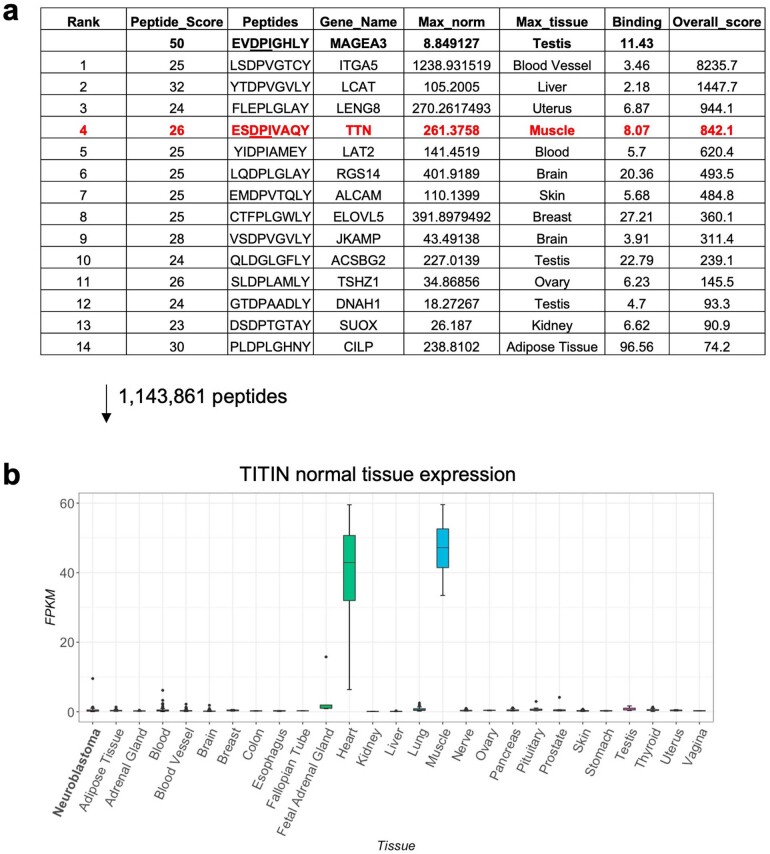
pMHC Cross-Reactivity Algorithm sCRAP Predicts MAGE-A3 toxicity through TITIN. **a**. Table of top predicted cross-reactive peptides to MAGE-A3 peptide EVDPIGHLY reveals cross-reactivity with Titin peptide ESDPIVAQY ranks 4^th^ out of 1,143,861 potential peptides presented on HLA-A*01:01. **b**. TITIN is highly expressed in GTEx RNA-sequencing of heart (n = 108) and muscle (n = 138) tissues. Lower and upper bounds of boxplot correspond to the first and third quartiles (the 25th and 75th percentiles); whiskers represent minima/maxima or 1.5*IQR.

To screen for PHOX2B peptide-specific clones, we adapted the Retained Display^33^ (ReD) system, a protein display platform that enables the flow cytometric selection of pMHC-binding scFvs in permeabilized bacterial cells, with a >10^11^-member scFv library. Clones that demonstrated apparent selectivity by flow cytometry were further tested against a panel of 95 unrelated peptides and 4 highly similar peptides presented on the same HLA allotype to select for clones with the highest selectivity (example of selective clone 10LH is shown in Fig. 2a). This selection step resulted in 25 scFv binders for screening in CAR T constructs. To address cross-reactivity with pMHC complexes in normal tissue, we developed an algorithm to predict potential selective cross-reactive antigen presentation (sCRAP; https://marisshiny.research.chop.edu/sCRAP/) on the same HLA allotype (Extended Data Fig. 12), which enabled pre-emptive selectivity filtering in early stages of scFv screening without the need of a previous alanine scan or receptor^34^. We benchmarked the sCRAP algorithm by testing its ability to predict the cross-reactivity of the MAGE-A3 peptide presented on HLA-A*01:01. The targeting of this peptide using an affinity-enhanced TCR previously resulted in fatal cross-reaction with another peptide derived from the TITIN protein presented on HLA-A*01:01 in myocardial tissue^12^. We predicted the cross-reactivity of MAGE-A3 with the TITIN peptide as the fourth ranked prediction out of 1,143,861 potential self-peptides presented in heart tissue (Extended Data Fig. 13).

We then screened our panel of PHOX2B-directed CARs against the top seven pMHC complexes predicted by sCRAP (Fig. 2b), which enabled us to eliminate cross-reactive CARs and to prioritize those with the highest degree of target selectivity (Fig. 2c). Out of 25 CARs screened, we selected clone 10LH, which had the highest specificity profile, for further development, showing only two peptides with >10% relative binding to 10LH compared with PHOX2B (Fig. 2d).

To test the functional significance of the binding to potential off-target pMHC complexes predicted by sCRAP, we pulsed HLA-matched, PHOX2B^−^ SW620 colon adenocarcinoma cells with the PHOX2B peptide and potential cross-reactive peptides across a range of concentrations. Pulsing with the PHOX2B peptide resulted in cytotoxicity when cultured together with 10LH at the lowest tested concentration of 0.1 µM. 10LH CAR T cells did not induce cytotoxicity with the most cross-reactive predicted peptide ABCA8 at 10 µM, and only induced killing at the supraphysiological concentration of 50 µM. The second most cross-reactive peptide with 10LH (MYO7B) showed no CAR cytotoxicity at concentrations up to 50 µM (Fig. 2e). Neither ABCA8 nor MYO7B has been detected in the normal tissue immunopeptidome^10^, and none of the peptides previously detected in the normal tissue immunopeptidome displayed any cross-reactivity with PC-CAR 10LH (Fig. 2d). These screens demonstrate the utility of sCRAP to pre-emptively identify off-target effects, efficiently screen their functional consequences and identify binders with highly selective binding to tumour targets.

The lead scFv 10LH bound the PHOX2B pMHC complex with a binding affinity (*K*_d_) of 13 nM and a slow off-rate (Fig. 2f; *K*_d_ = 7.6 × 10^−4^ s^–1^). We next performed an alanine scan for the 10LH CAR, characterizing binding to PHOX2B pMHC tetramers prepared with amino acid substitutions at each non-anchor position of the peptide^34^. The alanine scan revealed significant interactions of the PC-CAR receptor, with 5 out of 7 non-anchor residues of the PHOX2B peptide, including key residues protruding from the MHC cleft (interaction interface of 10LH with the PHOX2B pMHC complex mapped on crystal structures in Fig. 2h). This result highlights the improved selectivity of PC-CARs compared with the 3 or 4 residues that typically interact with the TCR^35^.

## PC-CAR T cells can recognize peptides across HLAs

**Fig. 3:**
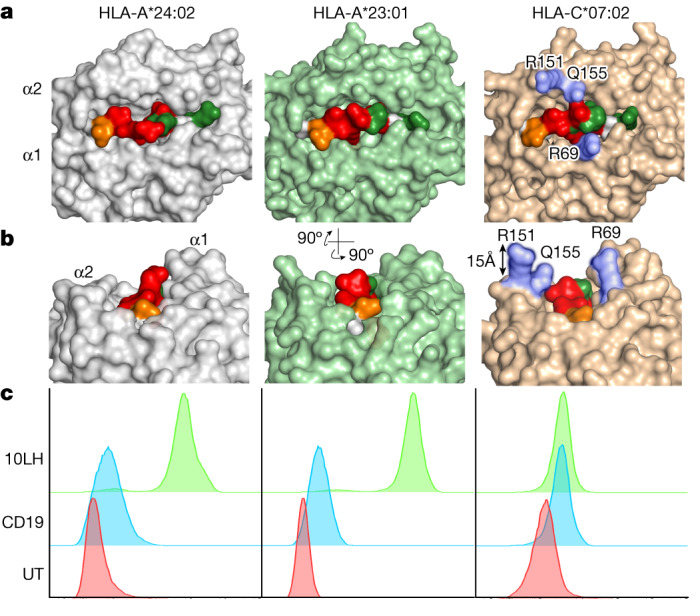
Structural basis of CARs binding the PHOX2B peptide presented on multiple HLAs. **a**, PHOX2B–HLA-A*2:024 crystal structure and models of PHOX2B in complex with HLA-A*23:01 and HLA-C*07:02. **b**, R151, Q155 and R69 charged and polar residues of HLA-C*07:02 align with key 10LH interaction residues I5, R6 and I7 (MHC residues in blue and PHOX2B–10LH interaction residues in red). R151, Q155 and R69 can create steric and charged hindrance of key peptide binding residues. **c**, Staining of PHOX2B PC-CAR 10LH (bottom) reveals binding to HLA-A*24:02 and HLA-A*23:01, but not to HLA-C*07:02. 10LH, PHOX2B PC-CAR; CD19, CD19-directed CAR; UT, untransduced T cells.

**Extended Data Fig. 14:**
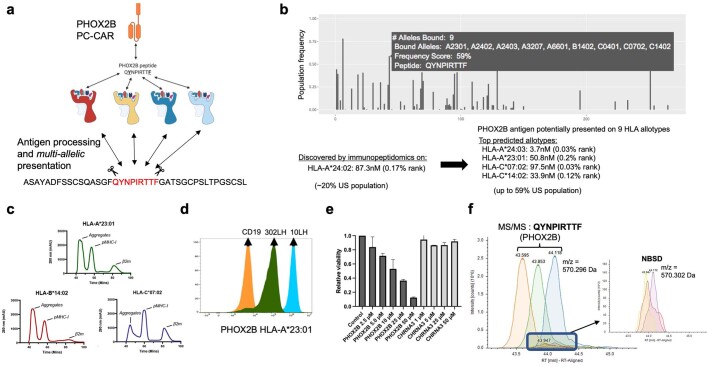
Presentation of the PHOX2B peptide by multiple HLA allotypes. **a**. Schematic of hypothesis proposing that PHOX2B peptide QYNPIRTTF detected by immunopeptidomics can be presented by additional HLA alleles after undergoing a common antigen processing pathway. **b**. Population-scale presentation across the length of the PHOX2B protein (all potential 9mers on x-axis) generated by ShinyNAP^8^ predicts PHOX2B peptide QYNPIRTTF to be presented by an additional 8 HLA alleles in addition to HLA-A*24:02. Additionally, QYNPIRTTF was found to bind additional common HLA alleles HLA-C*07:01, HLA-C*06:02, HLA-A*29:02, and HLA-A*32:01 using NetMHCpan 4.1^65^ and also predicted by HLAthena^44^. **c**. Size exclusion chromatography of PHOX2B peptide QYNPIRTTF refolded with HLA-A*23:01, HLA-B*14:02, and HLA-C*07:02 shows formation of stable pMHC complex with HLA-A*23:01 and HLA-C*07:02, and minimal complex with HLA-B*14:02. **d**. PC-CAR 10LH binds PHOX2B on HLA-A*23:01 demonstrates higher binding than 302LH, in concordance with observed in vivo activity (Fig. 4g). **e**. 10LH CAR kills HLA-A*23:01/PHOX2B^−^ WM873 cells when pulsed with PHOX2B peptide but not with CHRNA3 peptide n = 2 technical replicates; reported as mean +/− SD. **f**. Matched peptide search identifies unfragmented peaks in additional neuroblastoma tumors (NBSD shown). Unfragmented NBSD peaks are within 0.006 Da m/z of peaks in which PHOX2B peptide QYNPIRTTF was identified by MS/MS in other samples and eluted within one minute of fragmented peaks. While validated MS/MS peaks were found in 2/8 PDX tumors and 2/8 primary tumors, peaks with m/z and retention times matched to validated QYNPIRTTF peaks were identified in 6/8 PDX tumors and 7/8 primary tumors. Created with BioRender.com.

Given the prerequisite of antigen processing and presentation necessary for detection of a given MHC peptide by immunopeptidomics, we proposed that identical peptides could be presented on additional HLA allotypes capable of binding anchor residues of a peptide. Moreover, some of these peptide–HLA complexes could present a sufficiently similar molecular surface to be recognized by 10LH (Extended Data Fig. 14a). We tested this hypothesis using PHOX2B-specific CARs engineered to bind the PHOX2B 9-amino-acid-long fragment presented HLA-A*24:02 in a peptide-centric fashion. We used our population-scale antigen presentation tool ShinyNAP^8^ to identify additional HLA allotypes that could present the same PHOX2B peptide. The tool identified eight additional common HLAs predicted to bind the PHOX2B 9 amino acid residues (Extended Data Fig. 14b). We then used our pMHC complex structural modelling software RosettaMHC^36^ to model the structure of peptide complexes encompassing additional HLA alleles. The software identified HLA-A*23:01 as an additional top-scoring candidate for recognition by PC-CARs targeting the PHOX2B peptide originally discovered on HLA-A*24:02 (Fig. 3a,b). After validating stable complex formation between QYNPIRTTF and HLA-A*23:01 and HLA-C*07:02, and poor complex formation with HLA-B*14:02 by in vitro refolding with a synthetic peptide (Extended Data Fig. 14c), we measured the ability of 10LH to recognize these pMHC complexes using tetramer staining experiments. In addition to HLA-A*24:02, 10LH bound the PHOX2B 9-amino-acid-long QYNPIRTTF presented by HLA-A*23:01 (Fig. 3c). Moreover, although QYNPIRTTF formed a stable complex with HLA-C*07:02, the PHOX2B–HLA-C*07:02 tetramer did not bind 10LH CAR, which may result from presenting a distinct pMHC molecular surface in which side chains along the MHC α2-helix (R151 and Q155) protrude by 15 Å at the position axially aligned with the key 10LH binding residues of QYNPIRTTF (I5 and R6) (Fig. 3b). To demonstrate functionally relevant recognition of our prediction of PHOX2B presentation on HLA-A*23:01, we pulsed the HLA-A*23:01–PHOX2B^−^ melanoma cell line WM873 with the QYNPIRTTF peptide. Antigen-specific killing was induced in peptide-pulsed cells, whereas no cytotoxicity was induced in cells pulsed with a mismatched peptide (Extended Data Fig. 14d). HLA-A*23:01 is the most common non-A2 allele in people of African ancestry, which highlights the potential of PC-CARs to expand clinical application to underserved populations. Finally, we reanalysed our immunopeptidomics data, performing a matched peptide analysis to identify lower confidence potential peptide matches to QYNPIRTTF in additional samples in which the peak was not fragmented. We identified *m/z* matches within 1 min of retention time across 6 out of 8 PDX lines and 7 out of 8 patient samples, each expressing one of the HLA alleles predicted by our analyses. This result suggests that this peptide is ubiquitously expressed in neuroblastoma (Extended Data Fig. 14f). Together, these findings warrant additional investigation into targeting tumour self-antigens and neoantigens in the context of different HLA allotypes and demonstrate the potential to significantly expand the eligible patient population receiving peptide-centric scFv-based immunotherapies.

## PC-CAR T cells selectively eliminate tumours

**Fig. 4:**
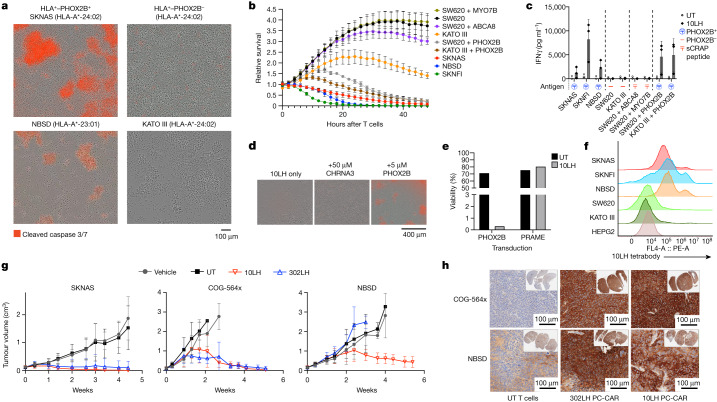
PHOX2B-specific PC-CAR T cells induce potent tumour killing in vitro and in vivo and kill different HLA allotypes. **a**–**c**, 10LH CAR induces specific killing and IFNγ release in neuroblastoma cells expressing HLA-A*24:02 and HLA-A*23:01 and PHOX2B (SKNAS, NBSD and SKNFI), but not in HLA-A*24:02–PHOX2B^−^ non-neuroblastoma tumour cells (SW620, HEPG2 and KATO III), unless PHOX2B peptide is added. No T cell activity was observed in SW620 cells when pulsed with 10 μM of predicted cross-reactive peptides ABCA8 or MYO7B (**b**,**c**). Cytotoxicity was visualized by T cell clustering and cleaved caspase in **a**, relative loss of confluence measured by loss of green fluorescence in GFP-transduced cancer cells in **b** and IFNγ release measured by ELISA in **c**. Assays performed using T cells from *n* = 3 donors, each in triplicate; plots presented as the mean ± s.d. **d**, Pulsing HLA-A*24:02–PHOX2B^−^ cell line SW620 with 5 μM PHOX2B induces complete cell killing when cultured with 10LH CAR, but no killing when pulsed with 50 μM CHRNA3. Repeated across three experiments with similar results. **e**, 10LH CAR specifically and specifically kills SW620 control cells transduced with PHOX2B, but not with PRAME. **f**, Staining cancer cells with tetramerized 10LH scFv enables the detection of PHOX2B pMHC on neuroblastoma cells but not in HLA-matched controls. **g**, PHOX2B-specific PC-CAR T cells induce potent tumour killing in mice engrafted with neuroblastoma PDX tumours, including the fast-growing line COG-564x and HLA-A*23:01 line NBSD. *n* = 6 mice enrolled per group (individual plots shown in Extended Data Fig. 17); data shown are representative from one of two independent, in vivo studies for each PDX line; shown as the mean ± s.d. **h**, Treatment with 10LH and 302LH PC-CARs potently upregulate HLA expression in PDX tumours collected from lone mice in each group reaching tumour burden compared with mice treated with untransduced T cells (COG-564x collected 11 days after treatment; NBSD collected 14 days after treatment for UT and 17 days after treatment for 10LH and 302LH; both tumours collected from one experiment).

**Extended Data Fig. 15:**
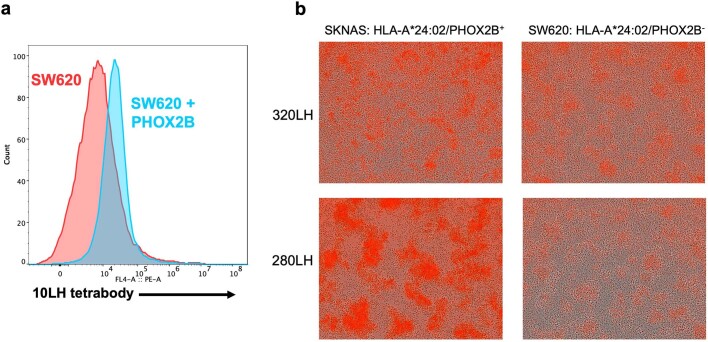
Tetramerized 10LH binds to PHOX2B pMHC. **a**. Pulsing SW620 HLA-A*24:02 with 50 μM PHOX2B peptide results in 10LH tetrabody binding. **b**. CARs predicted to be cross-reactive show killing in HLA-matched cells. CARs 320LH and 280LH predicted to cross-react with peptides presented on normal tissue demonstrate significant cross-reactivity in HLA-matched SW620 cells. Representative images shown from n = 3 technical replicates.

We next tested the on-target killing potential of 10LH using available HLA-A*24:02 and HLA-A*23:01 neuroblastoma cell lines (SKNAS, NBSD and SKNFI). Complete tumour cell killing and potent IFNγ release was induced after 24 h at an effector-to-target ratio of 5:1 (Fig. 4a–c and Supplementary Video 1). We tested the functional cross-reactivity of PC-CARs against the milieu of peptides presented by off-target tissues, and no activity occurred in three HLA-A*24:02 cell lines that do not express PHOX2B (SW620, a colorectal adenocarcinoma cell line; KATO III, a gastric adenocarcinoma cell line; and HEPG2, a hepatocellular carcinoma cell line) (Fig. 4a–c, Extended Data Fig. 10b and Supplementary Video 2). To validate the specificity of PC-CAR killing, we pulsed HLA-matched, PHOX2B^–^ cancer cell lines with the PHOX2B peptide and forcibly overexpressing PHOX2B. Specific killing occurred only in cells pulsed with the PHOX2B peptide and those transduced with full-length *PHOX2B* mRNA, and not in cells pulsed with a nonspecific CHRNA3, ABCA8 or MYO7B peptides presented on the same HLA, nor in cells transduced with full-length *PRAME* mRNA (Fig. 4b). This result demonstrates that native PHOX2B is processed and presented on MHC where it is specifically recognized by PC-CARs. To detect PHOX2B pMHC complexes on the cell surface, we generated a tetramerized 10LH scFv and stained on-target and off-target cell lines. The results showed significant surface PHOX2B pMHC in neuroblastoma cells and not in HLA-matched controls (Fig. 4f and Extended Data Fig. 15a), which suggested that these reagents have the potential to be used to assess the presence of antigen in biopsied tissue samples. Notably, CARs also flagged as cross-reactive by sCRAP demonstrated significant cross-reactivity, which validated the functional consequences of cross-reactivities predicted using our algorithm (Extended Data Fig. 15b).

**Extended Data Fig. 16:**
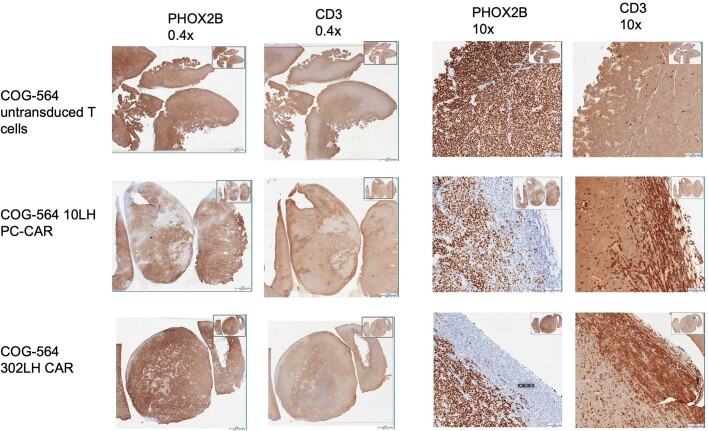
Immunohistochemistry of tumors collected from mice exceeding tumor burden. COG-564x tumor-bearing mice treated with 10LH and 302LH PC-CARs show T cell infiltration as measured by CD3, co-localized with loss of PHOX2B target expression and tissue necrosis. No evidence of T cell infiltration observed in mice treated with untransduced T cells. Tumors were collected from one of each mouse in treatment arms reaching tumor burden 11 days after receiving T cells. All other mice in treatment arms went on to achieve complete responses. Images shown from tumors collected from lone mice reaching tumor burden each study arm in single experiment.

**Extended Data Fig. 17:**
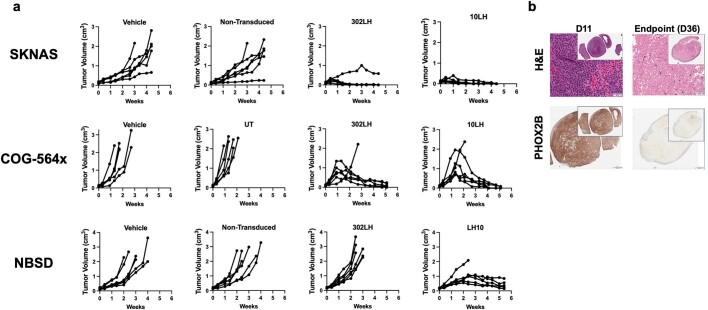
PC-CARs result in tumor ablation in mice engrafted with patient-derived xenografts. **a**. Tumor regression in individual mice in vivo treated with PC-CARs (n = 6; source data provided in Supplementary Table 3). **b**. COG-564x tumors collected from 302LH PC-CAR-treated mice collected at when mice reached tumor burden on day 11 (left) and endpoint of study on day 38 (right). Though tumors are detectable at endpoint, H&E and PHOX2B staining of 302LH PC-CAR-treated tumors reveals entirely necrotic tissue by endpoint of the study. Single available tumor collected from mouse reaching tumor burden for D11 and single endpoint tumor in one experiment collected for immunohistochemistry shown.

We next treated immunodeficient mice engrafted with HLA-A*24:02 (SKNAS and COG-564x) and HLA-A*23:01 (NBSD) xenografts with 10^6^ 10LH and 302LH transduced CAR T cells once tumours reached 100–250 mm^3^. Mice treated with 10LH or 302LH PC-CARs showed complete tumour responses in both HLA-A*24:02 xenografts (Fig. 4g). However, complete responses occurred only for the 10LH-treated mice with the HLA-A*23:01 NBSD xenografts. This correlated directly with the relative affinity of these two constructs against the PHOX2B peptide presented on HLA-A*23:01 (Extended Data Fig. 14c). This result suggests that a threshold affinity or distinct mode of binding by different scFvs may contribute to the ability to recognize the peptide in slightly altered conformations when presented by different HLA alleles. We also observed that CAR treatment induced substantial upregulation of MHC in tumours. The COG-564x PDX model was generated from a post-mortem blood-draw from a patient with high-risk *MYCN*-amplified neuroblastoma who had suffered multiple relapses. And tumours in this mouse model show an extremely rapid growth rate. In this experiment, one mouse treated with the 10LH construct had a tumour reach the end point size of 2 cm^3^ just 1 week after PC-CAR T cell therapy and was available for analysis, whereas all other tumours in this group nearly reached end point size and then all regressed (Fig. 4g and Extended Data Figs. 16 and 17). The lone COG-564x and NBSD tumours that reached end point showed significant PC-CAR T cell infiltrate and substantial upregulation of MHC expression compared with end point tumours treated with non-transduced CAR T cells (Fig. 4h and Extended Data Fig. 17b). This upregulation is probably due to the high levels of IFNγ release, as measured in vitro, which suggests that these therapies can activate T cell expansion at low antigen density to initiate a feed-forward cascade that increases MHC and antigen presentation.

## Discussion

Here we presented a process for identifying tumour-specific antigens derived from non-mutated oncoproteins, engineering PC-CARs against these tumour self-peptides and screening for cross-reactivity against MHC and the normal immunopeptidome. These methods resulted in PC-CARs that induced tumour cell killing across multiple HLA alleles in neuroblastoma and provide a roadmap for addressing the major challenges of therapeutic targeting of intracellular oncoproteins. These approaches demonstrate the value of pairing genomic, transcriptomic, epigenomic and immunopeptidomics datasets of normal and tumour tissues for the discovery of immunotherapy targets, and the utility of the ReD system paired with sCRAP to select ultra-rare scFv clones with desired binding and specificity profiles. Targeting of non-immunogenic self-antigens through pMHC-directed PC-CARs can expand the landscape of actionable immunotherapeutic targets and enable the development of personalized immunotherapies in neuroblastoma and other cancers. Owing to the limitations in ionization and canonical search spaces of our immunopeptidomics, the prioritized peptides described here are probably a fraction of the potential pMHC complexes available for immunotherapeutic targeting. In addition, neuroblastomas in general, and especially those harbouring *MYCN* amplification^37^, have a highly immunosuppressive tumour microenvironment such that future iterations of PC-CARs may require additional engineering to enable T cells to navigate to the pMHC complex target. However, our demonstration of significant upregulation of MHC (and therefore target) in our models may help alleviate this therapeutic obstacle.

We also highlighted the utility of pairing ShinyNAP with RosettaMHC to identify HLA allotypes capable of presenting identical peptides in a similar conformation. We suggest that these tools, in addition to matched peptide searches of immunopeptidomics data across multiple tumour samples, have the potential to appreciably expand the identification of tumour-specific peptides presented on multiple HLAs. We also showed that these algorithms generate incomplete predictions and that orthogonal methods are necessary for evaluating cross-HLA presentation and targeting. The potential ability to target these antigens beyond canonical HLA restriction can significantly increase the population of patients eligible to receive each PC-CAR construct and reach underserved populations, but this will need to be verified with other PC-CARs in development. The use of the sCRAP algorithm can rapidly exclude constructs with safety liabilities and prioritize those constructs with optimal safety profiles in contrast to alanine scanning, which determines construct-specific cross-reactivities post hoc^34^.

We demonstrated several distinct advantages of PC-CARs to target MHC peptides compared to TCRs. PC-CARs can be used to target essential unmutated oncoproteins by inducing immunogenicity using synthetic PC receptors, opening the possibility for higher degrees of peptide specificity and targeting through multiple HLAs. PC-CARs may also have an advantage over CARs that target membrane proteins in their ability to initiate a feed-forward loop of MHC upregulation and increased antigen density. These findings build on recent studies that demonstrate the ability to target neoantigens from mutated TP53 and RAS on MHC using scFv-based approaches and further demonstrate the utility of these approaches in targeting intracellular proteins^38,39^. We anticipate that the methods presented herein will facilitate the discovery of tumour-specific targets in other human cancers with high unmet need and envision a library of scFv-based synthetic immunotherapies that provides wider population-scale coverage of HLA genotypes for both neoantigens and self-antigens.

## Methods

### Neuroblastoma samples and cell lines

Five neuroblastoma CDX and three PDX that show a range of HLA expression by RNA sequencing and immunohistochemistry were selected for the initial immunopeptidomics experiment (Extended Data Table 1). All had whole exome sequencing and single nucleotide polymorphism array data available in addition to RNA sequencing^18^. Eight high-risk tumours with a mean mass of 0.56 g, ranging from 0.17 to 1.7 g, were obtained from the Children’s Oncology Group (COG; https://childrensoncologygroup.org/) with matched sequencing from Therapeutically Applicable Research To Generate Effective Treatments (TARGET; https://ocg.cancer.gov/programs/target). Informed consent from each research participant or legal guardian was obtained for each deidentified tumour and blood sample used in this study through the COG neuroblastoma biobanking study ANBL00B1.

Human-derived neuroblastoma cell lines, including SKNAS, SKNFI and NBSD, were obtained from the Maris Laboratory cell line bank. Neuroblastoma cell lines were cultured in RPMI supplemented with 10% FBS, 100 U ml^–1^ penicillin, 100 µg ml^–1^ streptomycin and 2 mM l-glutamine. Other human cancer cell lines, including Jurkat, SW620, HEPG2 and KATO III, were obtained from the American Type Culture Collection (ATCC). Jurkat cells were cultured in IMDM supplemented with 10% FBS, 100 U ml^–1^ penicillin, 100 µg ml^–1^ streptomycin and 2 mM l-glutamine. SW620 cells were cultured in RPMI supplemented with 10% FBS, 100 U ml^–1^ penicillin, 100 µg ml^–1^ streptomycin and 2 mM l-glutamine. HEPG2 cells were cultured in EMEM supplemented with 10% FBS, 100 U ml^–1^ penicillin, 100 µg ml^–1^ streptomycin and 2 mM l-glutamine. KATO III cells were cultured in IMDM supplemented with 20% FBS, 100 U ml^–1^ penicillin, 100 µg ml^–1^ streptomycin and 2 mM l-glutamine. Packaging cell lines, including platinum-A cells and HEK293T cells were obtained from Cell BioLabs and ATCC, respectively. Both packaging cell lines were cultured in DMEM supplemented with 10% FBS, 100 U ml^–1^ penicillin, 100 µg ml^–1^ streptomycin and 2 mM l-glutamine. All cell lines were grown under humidified conditions in 5% CO_2_ at 37 °C, and samples were regularly tested for mycoplasma contamination.

### Primary human T cells

Primary human T cells were obtained from anonymous donors through the Human Immunology Core at the University of Pennsylvania (Philadelphia, Pennsylvania) under a protocol approved by the Children’s Hospital of Philadelphia Institutional Review Board. Cells were cultured using AIM-V (Thermo Fisher Scientific) supplemented with 10% FBS, 100 U ml^–1^ penicillin, 100 µg ml^–1^ streptomycin and 2 mM l-glutamine under humidified conditions in 5% CO_2_ at 37 °C. T cell donors provided informed consent through the University of Pennsylvania Immunology Core.

### Isolation of HLA ligands by immunoaffinity purification

HLA class I molecules were isolated using standard immunoaffinity purification methods as previously described^1,2^. In brief, cell pellets were lysed in 10 mM CHAPS/PBS (AppliChem/Lonza) containing 1× protease inhibitor (Complete; Roche). Mouse MHC molecules were removed using a 1 h immunoaffinity purification method with H-2K-specific monoclonal antibody 20-8-4S, covalently linked to CNBr-activated sepharose (GE Healthcare). Remaining HLA molecules were purified overnight using the pan-HLA class I-specific monoclonal antibody W6/32 or a mix of the pan-HLA class-II-specific monoclonal antibody Tü39 and the HLA-DR-specific monoclonal antibody L243, covalently linked to CNBr-activated. pMHC complexes were eluted by the repeated addition of 0.2% trifluoroacetic acid (Merck). Elution fractions E1–E4 were pooled, and free MHC ligands were isolated by ultrafiltration using centrifugal filter units (Amicon, Merck Millipore). MHC ligands were extracted and desalted from the filtrate using ZipTip C18 pipette tips (Merck Millipore). Extracted peptides were eluted in 35 µl acetonitrile (Merck)/0.1% trifluoroacetic acid, centrifuged to complete dryness and resuspended in 25 µl 1% acetonitrile/0.05% trifluoroacetic acid. Samples were stored at −20 °C until analysis by LC–MS/MS.

### Analysis of HLA ligands by LC–MS/MS

Peptide samples were separated by reverse-phase LC (nanoUHPLC, UltiMate 3000 RSLCnano, Dionex) and subsequently analysed in an online coupled Orbitrap Fusion Lumos (Thermo Fisher Scientific). Samples were analysed in three technical replicates. Sample volumes of 5 µl (sample shares of 20%) were injected onto a 75 µm × 2 cm trapping column (Acclaim PepMap RSLC, Dionex) at 4 µl min^–1^ for 5.75 min. Peptide separation was subsequently performed at 50 °C and a flow rate of 300 nl min^–1^ on a 50 µm × 25 cm separation column (Acclaim PepMap RSLC, Dionex), applying a gradient ranging from 2.4 to 32.0% acetonitrile over the course of 90 min. Eluting peptides were ionized by nanospray ionization and analysed in a mass spectrometer implementing the TopSpeed method. Survey scans were generated in the Orbitrap at a resolution of 120,000. Precursor ions were isolated in quadrupole, fragmented by collision induced dissociation (CID) in the dual-pressure linear ion trap for MHC class I-purified peptides. Finally, fragment ions were recorded in the Orbitrap. An AGC target of 1.5 × 10^5^ and a maximum injection time of 50 ms was used for MS1. An AGC target of 7 × 10^4^ and a maximum injection time of 150 ms was used for MS2. The collision energy for CID fragmentation was 35%. For fragmentation, mass ranges were limited to 400–650 *m/z* with charge states 2+ and 3+ for MHC class I.

Synthetic peptides were analysed using a 30-min gradient owing to the simplicity of the sample. Full scans were acquired in the Orbitrap with a scan range of 300–1,200 at 120,000 resolution. The AGC target was 5.0 × 10^5^ with a maximum injection time of 50 ms. Precursor ions were isolated in quadrupole, fragmented (CID, HCD and ETD) and analysed in the Orbitrap. MS2 were also acquired in the Orbitrap with 30,000 resolution, collision energy of 35%, AGC of 5 × 10^4^ and maximum injection time of 150 ms. As the discovery analysis was completed using CID, synthetic peptides fragmented with CID were compared for validation.

The MS proteomics data have been deposited into the ProteomeXchange Consortium through the PRIDE^40^ partner repository with the dataset identifier PXD027182.

### HLA typing

FASTQ files from TARGET DNA and RNA sequencing data from cell lines were processed using the PHLAT algorithm, as previously validated on 15 HLA alleles^8^.

### Database search and spectral annotation

Data were processed against the human proteome per the Swiss-Prot database (https://www.uniprot.org, release 27 May 2021; 20,395 reviewed protein sequences contained) using the SequestHT algorithm^41^ in Proteome Discoverer (v.2.1; Thermo Fisher Scientific) software. Precursor mass tolerance was set to 5 ppm and the fragment mass tolerance to 0.02 Da. The search was not restricted to an enzymatic specificity. Oxidized methionine was allowed as a dynamic modification. FDR was determined using the Percolator algorithm based on processing against a decoy database consisting of shuffled sequences. FDR was set to 1%. Peptide lengths were limited to 8–14 amino acids for MHC class I. HLA annotation was performed using NetMHC-4.0 for HLA class I. For peptide matching, data were reprocessed using Proteome Discoverer (v.2.4; Thermo Fisher Scientific) using the same parameters but with the addition of the feature mapper node to allow peptide matching between samples. Synthetic peptides were searched using a similar approach, but Percolator was replaced with the fixed value PSM validator owing to the simplicity of the synthetic peptide sample. Gene ontology analyses were performed using PANTHER^42^ (http://geneontology.org/), and *P* values were calculated using Fisher’s exact test.

### HLA binding predictions

HLA binding predictions were performed using NetMHC (v.4.0)^43^, NetMHCpan (v.4.1)^44^ and HLAthena^45^ on HLA class I.

### scFv biopanning and CAR design

scFv binders against MHC-presented peptides were retrieved from a large (2 × 10^10^) naive phage display scFv library^46^. A competitive panning process was developed to identify specific binders targeting the pMHC complex based on previous protocols^47^. Biotinylated pMHC monomers (target antigens) and non-biotinylated tetramers (decoy competitors) were obtained from the NIH tetramer core facility. A total of 10^12^ copies of phages were depleted against magnetic beads (Invitrogen Dynabeads MyOne Streptavidin T1) for 30 min before incubation for 1.5 h with 5 µg biotinylated pMHC-conjugated beads in the presence of 20 µg irrelevant decoy competitors. After incubation, the beads were washed with PBS containing 0.05% Tween-20 (PBST buffer) 5 times followed by 2 PBS washes. The remaining bound phage were recovered by log-phase TG1 and rescued using the M13KO7 helper phage. The amplified phage was collected the next day by PEG–NaCl precipitation and used for the next round of panning. The target antigen input was decreased from 5 µg for the first round of panning to 2 µg and 0.5 µg for the second and third rounds, respectively, and the washing conditions were more stringent along with the panning rounds. After three rounds of panning, polyclonal phage ELISA was performed to evaluate enrichment. TG1 cells from the second and third rounds were randomly spotted into 96-well plates for soluble expression-based monoclonal ELISA as previously described^47,48^. Clones producing signals binding to target antigens but not the decoy competitors were amplified and sequenced. For protein preparation, these clones were transformed into HB2151 cells for expression, and proteins were purified by one-step Ni-NTA resin. Protein purity and homogeneity were analysed by SDS–PAGE. Protein concentration was measured spectrophotometrically (NanoVue, GE Healthcare). Second-generation CAR constructs were synthesized using scFv sequences with 4-1BB and CD3ζ co-stimulatory domains and cloned into a pMP71 vector for screening.

### ReD library panning

The Ruby scFv library (>10^11^ diversity) was constructed using fully germline IGLV3-1 and IGLV6-57 scaffolds paired with the IGHV3-23 scaffold, as previously described^33^, with fully synthetic amino acid diversity in both V_L_ and V_H_ CDR3 loops.

The Ruby library was panned for two rounds using PHOX2B(43–51)–MHC complex bound to MyOne Streptavidin C1 Dynabeads (Thermo Fisher, 65002). Panned library outputs were transferred into the ReD cell-display platform^33^ and cells were permeabilized using 0.5% *n*-octyl β-d-thioglucopyranoside (Anatrace, 0314) and labelled using recombinant PHOX2B pMHC complex ligated to fluorophores excitable by 405 nm and 488 nm lasers. Cells that were positive for target binding were isolated using a FACSMelody sorter (Becton-Dickinson).

After two rounds of positive selection for binding to the PHOX2B–MHC complex, two further FACS rounds were conducted using counter-labelled A*24:02–MHC complexes with unrelated peptides. After four rounds of FACS, individual colonies were picked and grown in 96-well plates before scFv induction, cell permeabilization and PHOX2B–MHC labelling and detection by CytoFLEX (Beckman Coulter).

Clones that were identified as binding specifically to the PHOX2B(43–51)–MHC complex were sequenced, and unique scFvs were expressed as fusions to the AviTag biotinylation motif in *Escherichia coli*. Biotinylated scFv protein was released through permeabilization with 0.5% *n*-octyl β-d-thioglucopyranoside and purified to about 90% purity on Nickel NTA agarose resin (ABT, 6BCL-NTANi).

### Binding kinetics

Affinity measurements were performed using a BLItz system (ForteBio) and analysed using BLItz Pro software. Streptavidin biosensors (ForteBio, 18–5019) were loaded with AviTag-biotinylated scFv, blocked with biotin, washed in PBS and then associated with pMHC ligand in PBS.

### Steady-state binding assay

An equilibrium binding assay to target pMHC complexes was also established using MyOne Streptavidin C1 Dynabeads. In brief, 50 µg of Streptavidin C1 Dynabeads were incubated with excess biotinylated scFv before being blocked with free biotin and washed in PBS. Fluorophore-labelled pMHC complex was added to a concentration of 3.5 nM and incubated for 1 h at 4 °C followed by 10 min at 25 °C. Binding of the free MHC complex to the beads was quantitated using CytoFLEX at 488 nm (excitation) and 525 nm (emission). Binding was normalized to beads without scFv and with unrelated control MHC complex.

This bead-binding assay was used to quantitate the binding of scFv to MHC complexes with alanine-scan substitutions of the PHOX2B peptides and to a plate of 95 unrelated 9-amino-acid peptide A*24:02–MHC complexes. The degree of cross-reactivity of binding of MHC complexes with peptides identified as having high homology to the PHOX2B peptide was analysed using eXpitope 2.0.

### Viral production and transduction of Jurkat and primary T cells

Retrovirus for transduction of Jurkat cells and primary CD4/8T cells was produced using platinum-A cells, a retroviral packaging cell line. Cells were plated in 6-well plates at 7 × 10^5^ cells per well and transfected with 2.5 µg of the appropriate TCR or CAR construct in the retroviral vector pMP71 using Lipofectamine 3000 (Life Technologies, Invitrogen). After 24 h, medium was replaced with IMDM-10% FBS or AIM-V-10% FBS for Jurkat cells or primary cells, respectively. Supernatants were collected and filtered through 0.2 μm filters after 24 h of incubation.

A second-generation lentiviral system was used to produce replication-deficient lentivirus. The day preceding transfection, 15 million HEK293T cells were plated in a 15-cm dish. On the day of transfection, 80 µl Lipofectamine 3000 (Life Technologies, Invitrogen) was added to 3.5 ml room-temperature Opti-MEM medium (Gibco). Concurrently, 80 µl P3000 reagent (Thermo Fisher Scientific), 12 µg psPAX2 (Gag/Pol), 6.5 µg pMD2.6 (VSV-G envelope) and a matching molar quantity of transfer plasmid were added to 3.5 ml room-temperature Opti-MEM medium. Virus supernatant was collected after 24 and 48 h and briefly centrifuged at 300*g* and passed through a 0.45 µm syringe.

Jurkat cells were plated in 6-well plates pre-treated with 1 ml well/retronectin (20 mg ml^–1^, Takara) at 1 × 10^6^ cells per well and spinoculated with 2 ml retroviral supernatant at 800*g* for 30 min at room temperature. After 24 h, cells were collected and grown in IMDM with 10% FBS.

Primary T cells were thawed and activated in culture for 3 days in the presence of 100 U ml^–1^ IL-2 and anti-CD3/CD28 beads (Dynabeads, Human T-Activator CD3/CD28, Life Technologies) at a 3:1 bead:T cell ratio. On days 4 and 5, activated cells were plated in 6-well plates pre-treated with 1 ml well/retronectin (20 mg ml^–1^, Takara) at 1 × 10^6^ cells per well and spinoculated with 2 ml retroviral supernatant at 2,400 r.p.m. for 2 h at 32 °C. On day 6, cells were collected and washed, beads were magnetically removed, and cells were expanded in AIM-V and 10% FBS supplemented with 25 U ml^–1^ IL-2.

Primary human T cells were thawed and activated in culture for 1 day in the presence of 5 ng ml^–1^ recombinant IL-7, 5 ng ml^–1^ recombinant IL-15 and anti-CD3/CD28 beads (Dynabeads, Human T-Activator CD3/CD28, Life Technologies) at a 3:1 bead:T cell ratio in G-Rex system vessels (Wilson Wolf). On day 2, thawed lentiviral vector was added to cultured T cells with 10 µg ml^–1^ polybrene (Millipore Sigma), and 24 h later, vessels were filled with complete AIM-V medium supplemented with indicated concentrations of IL-7 and IL-15. On day 10, cells were collected and washed. Activation beads were magnetically removed, and cell viability was determined before freezing.

Human neuroblastoma cell lines were plated in 6-cm dishes, and 2 ml of thawed lentiviral vector produced with transfer plasmid pLenti-CMV-eGFP-Puro (Addgene, plasmid 17448) was added with 10 µg ml^–1^polybrene (Millipore Sigma). Cells were selected for eGFP expression using flow-assisted cell sorting (BD FACSJazz, BD Biosciences) followed by 10 µg ml^–1^ puromycin selection.

### sCRAP prediction

Tumour antigens were compared against the entire normal human proteome on the matched HLA (85,915,364 total normal peptides among HLA 84 HLAs). Each residue in the same position of the tumour and human peptides was assigned a score for perfect match, similar amino acid classification or different polarity, scoring 5, 2 or –2, respectively (Extended Data Fig. 12). Similarity scores were calculated based on amino acid classification and hydrophobicity was determined using residues one and three until eight and excluding MHC anchor residues. Next, the maximum normal tissue RPKM values were identified from 1,643 normal tissues in GTEx. Normal peptides were compared with a database of normal tissue immunopeptidomes^49^. The overall cross-reactivity score for each normal peptide was then calculated using the following equation:

$$\frac{{\sum }_{i=3}^{n}{P}_{i}}{b\times {E}_{\max }}$$

where *n* is the peptide length, *P* is the score of each amino acid of the normal peptide compared to the tumour antigen, *b* is the pMHC binding affinity of the normal peptide, and *E*_max_ is the maximum normal tissue expression. The algorithm is available at https://marisshiny.research.chop.edu/sCRAP.

### Tetramer and dextramer staining and flow cytometric analysis

Surface expression and binding of TCR-transduced and CAR-transduced Jurkat cells and primary T cells was measured by staining with PE-conjugated or APC-conjugated dextramers carrying NB antigen pMHC (Immudex). Cells were collected from culture, washed with 2 ml PBS at 800*g* for 5 min, incubated with 1 µl dextramer for 10 min in the dark, washed again and resuspended in 300 µl PBS for analysis. Typically, 5 × 10^5^ cells were used for staining and analysed on a BD LSR II (BD Biosciences) or an Attune Acoustic Focusing cytometer (Applied Biosystems, Life Technologies). Flow cytometry data were collected using CytExpert (Beckman Coulter) and FACSDiva (BD Biosciences). The gating strategy for all tetramer and dextramer staining is shown in Extended Data Fig. 9f.

### Cross-reactivity pMHC screen

Potential cross-reactive peptides (GenScript) were suspended at a 200 µM working concentration. For each test, 0.5 µl of peptide was added to 5 µl HLA-A*24:02 empty loadable tetramer (Tetramer Shop) before incubating on ice for 30 min or using TAPBR peptide exchange as previously described^50^. Following preparation, pMHC tetramers were used to stain cells (described above). CAR construct cross-reactivity values were determined using Jurkat cells transduced with CAR clones followed by staining with HLA-A*24:02 tetramers loaded with cross-reactive peptides. Mean fluorescent intensity was compared across peptides to determine cross-reactivity.

### Antigen-specific CD8 T cell enrichment and expansion

Normal donor monocytes were plated on day 1 in 6-well plates at 5 × 10^6^ per well in RPMI-10 FBS supplemented with 10 ng ml^–1^ IL-4 (Peprotech) and 800 IU ml^–1^ GM-CSF (Peprotech) and incubated at 37 °C overnight. On day 2, fresh medium supplemented with 10 ng ml^–1^ IL-4 and 1,600 IU ml^–1^ GM-CSF was added to the monocytes and incubated at 37 °C for another 48 h. On day 4, non-adherent cells were removed, and immature dendritic cells washed and pulsed with 5 µM peptide in AIM-V-10% FBS supplemented with 10 ng ml^–1^ IL-4, 800 IU ml^–1^ GM-CSF, 10 ng ml^–1^ LPS (Sigma-Aldrich) and 100 IU ml^–1^ IFNγ (Peprotech) at 37 °C overnight. Day 1 was repeated on days 4 and 8 to generate dendritic cells for the second and third stimulations on days 8 and 12, respectively.

On day 5, normal donor-matched CD8^+^ T cells were enriched using the protein kinase inhibitor dasatinib (Sigma-Aldrich), dextramers and anti-PE or anti-APC beads (Miltenyi Biotec) as previously described^51^. Enriched T cells were co-incubated with the appropriate pulsed dendritic cells in AIM-V-10% FBS. Day 5 protocol was repeated on day 8 and day 12 using dendritic cells generated on days 4 and 8 for the second and third stimulation, respectively. Expanded T cells were validated for antigen-specificity by staining with the appropriate dextramers and for activation marker 41BB/CD137 (BioLegend).

### Antigen-specific T cell sorting, sequencing and cloning

Expanded T cells were stained with CD3, CD8, CD14, CD19, live/dead, and matched and mismatched dextramer, and single-antigen-specific T cells were sorted using a FACSAria Fusion (BD Biosciences).

Sorted cells were loaded onto 10x Genomics 5′ V(D)J chips and libraries prepared according to manufacturer’s protocols. TCRα/β amplicons were run on MiSeq using 5,000 reads per cell. Sequencing data were processed using Cell Ranger and analysed using Loupe VDJ Browser. TCRα and β chains were codon optimized and synthesized into bicistronic expression cassettes using engineered cysteine residues in the TCR constant domains, using F2A ribosomal skip sites and furin cleavage sites^52^. TCR cassettes were cloned into pMP71 retroviral vector.

### Incucyte cytotoxicity assay

A total of 0.5 × 10^5^ tumour cell targets were incubated with varying ratios of transduced primary cells (5 × 10^5^, 2.5 × 10^5^, 1 × 10^5^, 0.5 × 10^5^ and 2.5 × 10^4^ for 10:1, 5:1, 2:1, 1:1 and 1:2 effector-to-target ratios, respectively) in 96-well plates at 37 °C in the presence of 0.05 µM caspase-3/7 red (Incucyte, Essence BioScience). Plates were run on an Incucyte Zoom or S3 for 24–72 h and measured for apoptosis activity through caspase cleavage and comparison of relative confluency. Following the assay, supernatants were collected for ELISA. Total GFP integrated intensity (total GCU × μm^2^ per image) was assessed as a quantitative measure of live, GFP^+^ tumour cells. Values were normalized to the *t* = 0 measurement.

### Cytokine secretion assays

Cell supernatant collected from cell cytotoxicity assays was thawed and plated in triplicate for each condition. IFNγ and IL-2 levels were determined using ELISA kits according to the manufacturer’s protocol (BioLegend).

### Antigen processing and presentation

Neuroblastoma cell lines were titrated with H1N5 influenza virus and infectivity was measured by flow cytometry using virus nucleoprotein (NP) antibody. HLA-A2 neuroblastoma cell lines were cultured with either 5 μM CEF1 or 50 HAU of H1N5 virus then cultured with M1 antigen-specific T cell hybridoma provided by D. Canaday^53^. T cell activation was measured using IL-2 ELISA (Abcam).

### Expression, refolding and purification of recombinant pHLA molecules

HLA-A*02:01, HLA-A*24:02:01, HLA-A*23:01, HLA-B*14:02 and HLA-C*07:02 constructs for bacterial expression were cloned into pET24a+ plasmids. DNA plasmids encoding HLA heavy chain and human β2M (light chain) were transformed into *E.* *coli* BL21-DE3 (Novagen), expressed as inclusion bodies and refolded using previously described methods^54^. *E.* *coli* cells were grown in autoinduction medium for (16–18 h)^55^. Afterwards, the *E.* *coli* cells were collected by centrifugation and resuspended with 25 ml BugBuster (Milipore Sigma) per litre of culture. The cell lysate was sonicated and subsequently pelleted by centrifugation (5,180*g* for 20 min at 4 °C) to collect inclusion bodies. The inclusion bodies were washed with 25 ml of wash buffer (100 mM Tris pH 8.0, 2 mM EDTA and 0.01% v/v deoxycholate), sonicated and pelleted by centrifugation. A second wash was done using 25 ml Tris-EDTA buffer (100 mM Tris pH 8.0 and 2 mM EDTA). The solution was once again resuspended by sonication then centrifuged. The inclusion bodies were then solubilized by resuspending in 6 ml of resuspension buffer (100 mM Tris pH 8.0, 2 mM EDTA, 0.1 mM DTT and 6 M guanidine-HCl). Solubilized inclusion bodies of the heavy and light chain were mixed in a 1:3 molar ratio and then added dropwise over 2 days to 1 litre of refolding buffer (100 mM Tris pH 8.0, 2 mM EDTA, 0.4 M arginine-HCl, 4.9 mM l-glutathione reduced, and 0.57 mM l-glutathione oxidized) containing 10 mg of synthetic peptide at >98% purity confirmed by MS (Genscript). Refolding was allowed to proceed for 4 days at 4 °C without stirring. Following this incubation period, the refolding mixture was dialysed into size-exclusion buffer (25 mM Tris pH 8.0 and 150 mM NaCl). After dialysis, the sample was concentrated first using a Labscale Tangential Flow Filtration system and then using an Amicon Ultra-15 Centrifugal 10 kDa MWCO Filter Unit (Millipore Sigma) to a final volume of 5 ml. Purification was performed using size-exclusion chromatography on a HiLoad 16/600 Superdex 75 column. The purified protein was exhaustively exchanged into 20 mM sodium phosphate pH 7.2 and 50 mM NaCl. The final sample was validated using SDS–PAGE to confirm the formation of a pMHC complex containing both the heavy and light chains.

### Differential scanning fluorimetry

To measure the thermal stability of the pMHC class I molecules, 2.5 μM protein was mixed with 10× Sypro Orange dye in matched buffer (20 mM sodium phosphate pH 7.2, 100 mM NaCl) in MicroAmp Fast 96well plates (Applied Biosystems) at a final volume of 50 μl. Differential scanning fluorimetry was performed using an Applied Biosystems ViiA qPCR machine with excitation and emission wavelengths at 470 nm and 569 nm, respectively. Thermal stability was measured by increasing the temperature from 25 °C to 95 °C at a scan rate of 1 °C min^–1^. Melting temperatures (*T*_m_) were calculated in GraphPad Prism 7 by plotting the first derivative of each melt curve and taking the peak as the *T*_m_.

### Protein crystallization

Purified HLA-A*02:01–LLLPLLPPL, HLA-A*02:01–LLPLLPPLSP, HLA-A*02:01–LLPLLPPLSPS, HLA-A*02:01–LLPRLPPL and HLA-A*24:02–QYNPIRTTF complexes were used for crystallization. Proteins were concentrated to 10–12 mg ml^–1^ in 50 mM NaCl, 25 mM Tris pH 8.0, and crystal trays were set up using a 1:1 protein-to-buffer ratio at room temperature. Optimal crystals for HLA-A*02:01–LLLPLLPPL, HLA-A*02:01–LLPLLPPLSP and HLA-A*02:01–LLPLLPPLSPS were obtained with 1 M sodium citrate dibasic and 0.1 M sodium cacodylate pH 6.5. For HLA-A*02:01–LLPRLPPL, diffracting crystals were obtained with 0.2 M magnesium chloride, 0.1 M HEPES pH 7.0 and 20% PEG 6000. HLA-A*24:02–QYNPIRTTF diffracting crystals were obtained with 0.1 M HEPES pH 7.0 and 10% PEG 6000. Diffraction-quality crystals were collected and incubated from the above conditions plus glycerol as a cryoprotectant and flash-frozen in liquid nitrogen before data collection. All crystals used in this study were grown using the hanging drop vapour diffusion method. Data were collected from single crystals under cryogenic conditions at Advanced Light Source (beam lines 8.3.1 and 5.0.1). Diffraction images were indexed, integrated and scaled using MOSFLM and Scala in CCP4 Package^56^. Structures were determined by Phaser^57^ using previously published structures of HLA-A*02:01 (PDB identifier 5C07)^58^ and HLA-A*24:02 (PDB identifier 3VXN)^59^. Model building and refinement were performed using COOT^60^ and Phenix^61^, respectively.

### Homology modelling of pHLA complexes using RosettaMHC

Three-dimensional structural models of HLA-A*23:01 and HLA-C*07:02 bound to the peptide QYNPIRTTF were generated using RosettaMHC, an in-house method for modelling the α_1_/α_2_ peptide binding domains of pMHC class I molecules^36^. In brief, the amino acid sequences of HLA-A*23:01 and HLA-C*07:02 were first obtained from the IPD-IMGT/HLA Database^62^. The sequence of HLA alleles of interest was aligned against the sequences of 318 HLA curated template structures available in RosettaMHC. For each allele, all candidate templates were selected according to a 70% sequence identity criterion between aligned residues within the peptide-binding groove (within 3.5 Å of any peptide heavy atom). Generation of 3D models was performed using a Monte Carlo sampling of sidechain rotamer conformations, followed by gradient-based optimization of all backbone and side chain degrees of freedom. For each pHLA complex, the top five models with the lowest Rosetta binding energy were selected as the final structural ensemble. The quality of the final models was assessed using the Molprobity webserver^63^. Analysis of polar contacts and surface area were performed using the PyMOL Molecular Graphics System (v.2.4.1).

### Immunohistochemistry

CD3 (Dako A0452), PHOX2B (Abcam ab183741) and HLA-ABC (Abcam ab70328) antibodies were used to stain formalin-fixed paraffin-embedded tissue slides. Staining was performed on a Bond Max automated staining system (Leica Biosystems). A Bond Refine polymer staining kit (Leica Biosystems, DS9800) was used. The standard protocol was followed with the exception of the primary antibody incubation, which was extended to 1 h at room temperature. CD3, PHOX2B and HLA-ABC antibodies were used at 1:100, 1:500 and 1:1,200 dilutions, respectively. Antigen retrieval was performed with E1 (Leica Biosystems) retrieval solution for 20 min (E2 for PHOX2B). Slides were rinsed, dehydrated through a series of ascending concentrations of ethanol and xylene, then coverslipped. Stained slides were then digitally scanned at ×20 magnification on an Aperio CS-O slide scanner (Leica Biosystems).

### Mouse PC-CAR T cell preclinical trials

NOD SCID Gamma (NSG) female (6–8 weeks of age) mice from Jackson Laboratory (stock number 005557) were used to propagate subcutaneous xenografts. All mice were maintained under barrier conditions and experiments were conducted using protocols and conditions approved by the Institutional Animal Care and Use Committee at the Children’s Hospital of Philadelphia. Treatment was initiated through lateral tail intravenous injection. The dose administered was 100 µl per animal of vehicle or CAR T cells as a single treatment. Treatment was administered at weeks 8–10 when tumour volumes reached 150–250 mm^3^. Six mice were enrolled per group based on previous experience and randomized based on tumour size. The mouse technician was blinded to T cell engineering. Tumour volume and survival were monitored through bi-weekly measurements until the tumours reached a size of 2.0 cm^3^ or mice showed signs of graft versus host disease (GVHD). Animals were removed from study and studies terminated following onset of GVHD when animals display hunched posture, rapid breathing, urine staining, weight loss and a body condition score of 2, as determined by visual inspection. Onset of GVHD defined as urine staining and weight loss of 20% or weight loss of 10–15% if accompanied by hunched posture, laboured breathing or poor body condition.

### Statistics and reproducibility

Box and whisker plot representations of data show the median as centre, 25th percentile and 75th percentile as bounds of boxes for plots shown in Fig. 1e and Extended Data Figs. 1 and 13b.

### Reporting summary

Further information on research design is available in the Nature Portfolio Reporting Summary linked to this article.

## Online content

Any methods, additional references, Nature Portfolio reporting summaries, source data, extended data, supplementary information, acknowledgements, peer review information; details of author contributions and competing interests; and statements of data and code availability are available at 10.1038/s41586-023-06706-0.

### Supplementary information

Supplementary Fig. 1 The original source images for all data obtained by immunoblotting that show the uncropped form of the gels shown in Extended Data Fig. 10. Gels are labelled according to loading control (Ku80) or experimental samples (PHOX2B).

Reporting Summary

Supplementary Table 1 **Immunopeptidomics data for neuroblastoma PDX/CDX**. Immunopeptidomics data for neuroblastoma PDX/CDX and primary patient tumours. LC–MS/MS proteomics for HLA class I peptides eluted from eight PDX/CDX and eight primary patient tumours using 1% FDR. Peptides are annotated by source protein/gene, tumour line, best predicted HLA binder based on patient HLA class I allotype and predicted binding affinity/rank.

Supplementary Table 2 **Annotation of immunopeptidomics data**. Annotation of raw sample files uploaded on PRIDE (https://www.ebi.ac.uk/pride/archive/projects/PXD027182).

Supplementary Table 3 **Source data for mouse tumour measurements**. Source data for mouse tumour measurements shown in Fig. 4g and Extended Data Fig. 17.

Supplementary Video 1 **10LH PC-CAR cytotoxicity assay with on-target SKNAS cells**. 10LH CAR induces specific killing in SKNAS HLA-A*24:02–PHOX2B^+^ neuroblastoma cells at 5:1 effector-to-target ratio. Cytotoxicity visualized by T cell clustering and cleaved caspase.

Supplementary Video 2 **10LH PC-CAR cytotoxicity assay with off-target SW620 cells**. 10LH CAR does not induce cell killing in SW620 HLA-A*24:02–PHOX2B^–^ cells expressing at 5:1 effector-to-target ratio. Cytotoxicity visualized by T cell clustering and cleaved caspase.

## Figures and Tables

**Extended Data Table 1:**
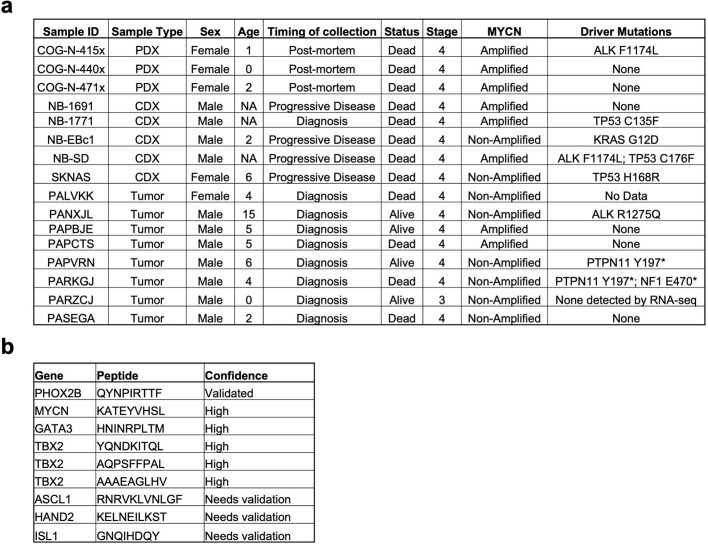
Antigen discovery strategies in neuroblastoma

**a**. Annotation of CDX (cell line derived xenograft), PDX (patient derived xenograft), and high-risk primary patient tumors selected for immunopeptidomics. Age in years. Stage 4 = metastatic at diagnosis, Stage 3 = large unresectable primary tumor but no hematogenous metastases. Protein coding mutations in known neuroblastoma oncogenes. **b**. Peptides derived from CRC genes detected in neuroblastoma immunopeptidome. High confidence indicates spectra were detected in multiple replicates and possess high coverage across b and y ions. Spectra containing peptides from ASCL1, HAND2, and ISL1 were observed in at least one replicate and/or with partial coverage across B and Y ions, necessitating further validation to confirm presentation of peptides.

## Data Availability

Neuroblastoma HLA class I immunopeptidomics data are available through PRIDE (accession PXD027182). Sample files are annotated in Supplementary Table 2. All proteins structures are available in the PDB under accession codes 7MJ6 (HLA-A*02:01–LLLPLLPPL), 7MJ7 (HLA-A*02:01–LLPLLPPLSP), 7MJ8 (HLA-A*02:01–LLPLLPPLSPS), 7MJ9 (HLA-A*02:01/LLPRLPPL) and 7MJA (HLA-A*24:02–QYNPIRTTF). The sCRAP algorithm is accessible to the scientific community through a web portal (https://marisshiny.research.chop.edu/sCRAP/). All other data are available within the article and supplementary information files or by request from the corresponding author.
