# Supplementary material for: Targeting of intracellular oncoproteins with peptide-centric CARs
Source: Nature. 2023 Nov 8;623(7988):820–7. doi: 10.1038/s41586-023-06706-0 (PMC10665195; doi:10.1038/s41586-023-06706-0)
Supplement: Supplementary file 1 — The original source images for all data obtained by immunoblotting that show the uncropped form of the gels shown in Extended Data Fig. 10. Gels are labelled according to loading control (Ku80) or experimental samples (PHOX2B). [file 41586_2023_6706_MOESM1_ESM.pdf]

nature portfolio
https://doi.org/10.1038/s41586-023-06706-0

Supplementary information

Targeting of intracellular oncoproteins with peptide-centric CARs

In the format provided by the authors and unedited

Raw Gel Images

Ku80 blot

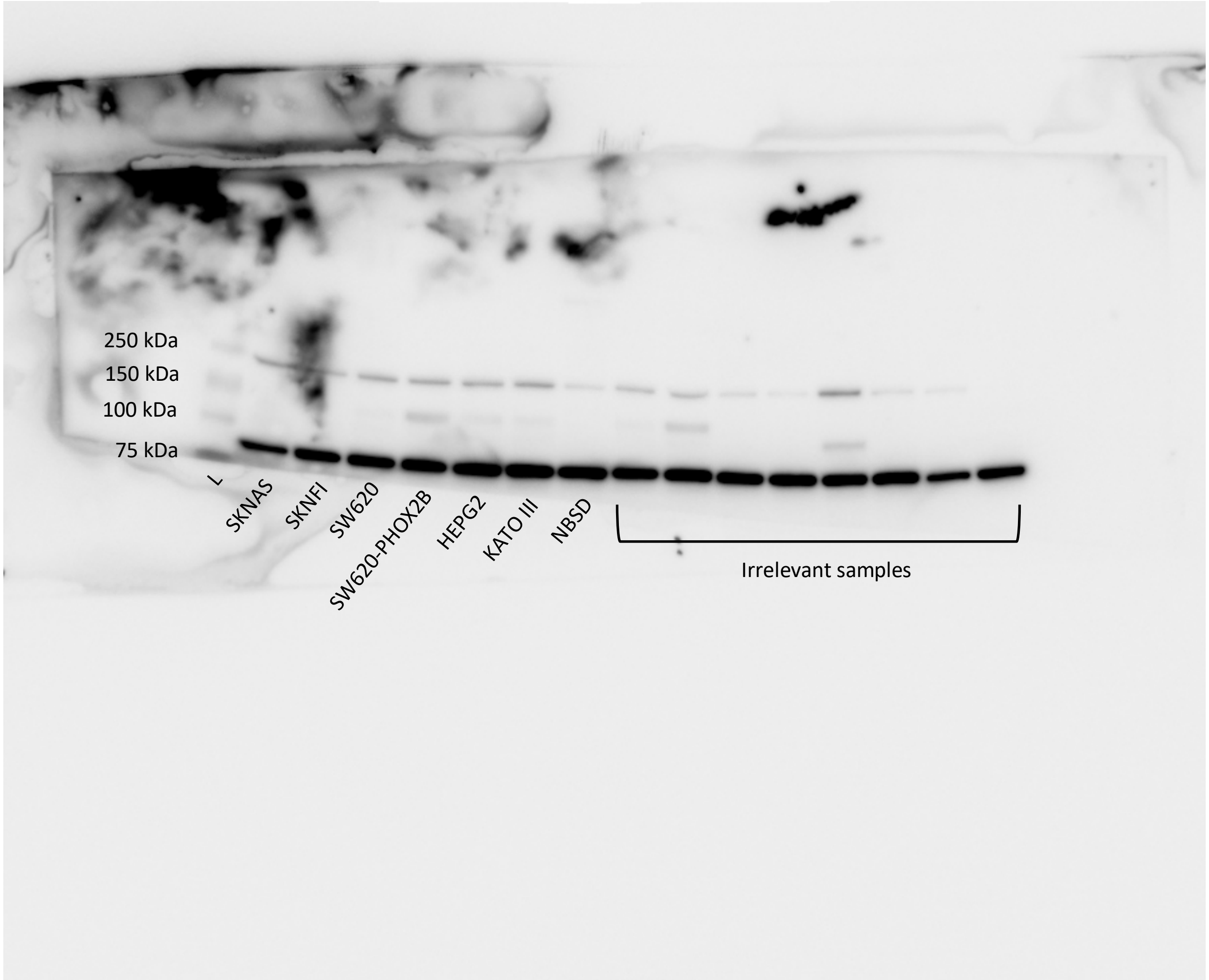

PHOX2B blot

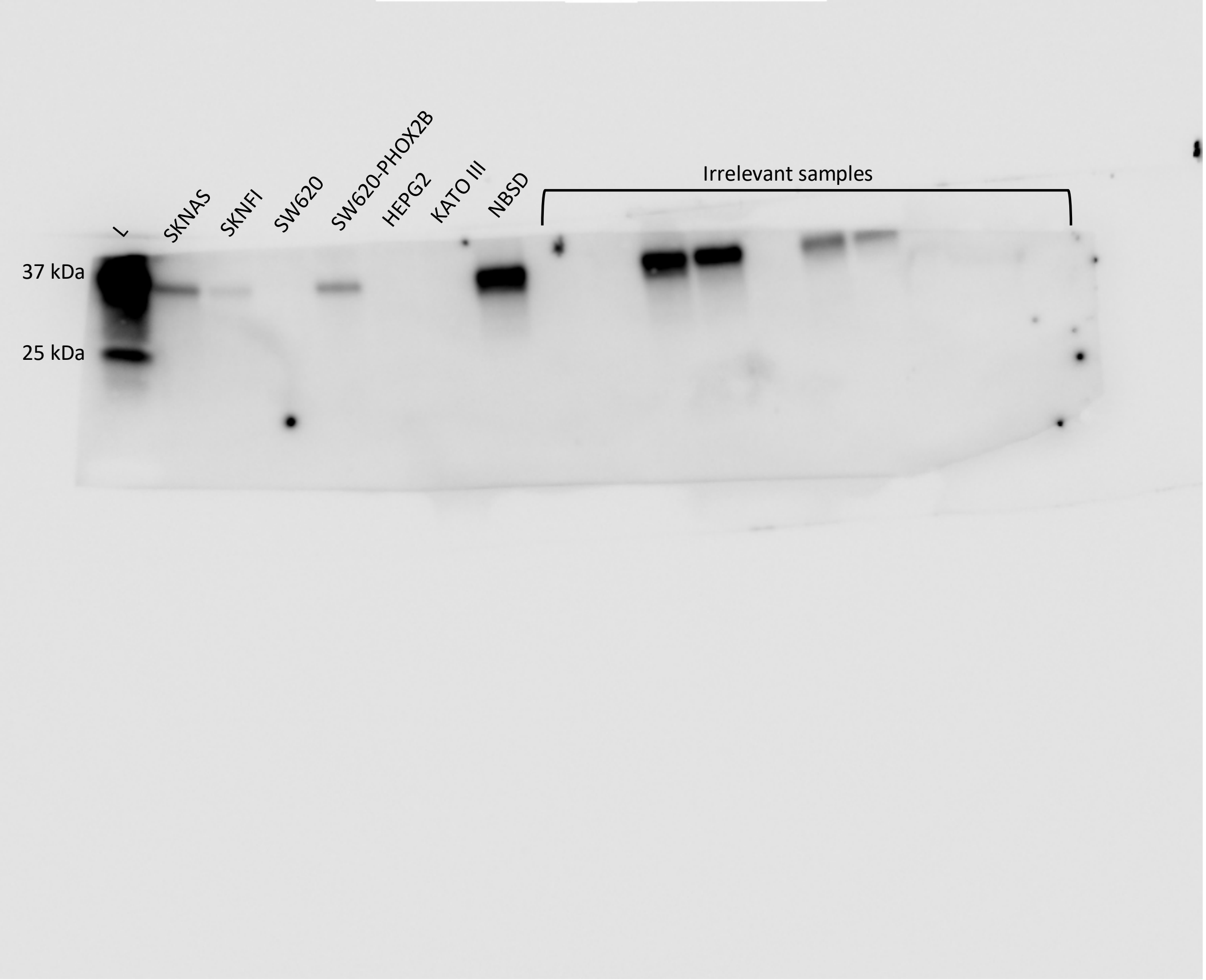
L
SKNAS
SKNFI
SW620
SW620-PHOX2B
HEPG2
KATO III
NBSD
Irrelevant samples
37 kDa
25 kDa
