# Supplementary material for: Targeting of intracellular oncoproteins with peptide-centric CARs
Source: Nature. 2023 Nov 8;623(7988):820–7. doi: 10.1038/s41586-023-06706-0 (PMC10665195; doi:10.1038/s41586-023-06706-0)
Supplement: Supplementary file 2 — Reporting Summary [file 41586_2023_6706_MOESM2_ESM.pdf]

nature portfolio

Corresponding author(s): John M. Maris

Last updated by author(s): Jul 23, 2023

Reporting Summary

Nature Portfolio wishes to improve the reproducibility of the work that we publish. This form provides structure for consistency and transparency in reporting. For further information on Nature Portfolio policies, see our Editorial Policies and the Editorial Policy Checklist.

Statistics

For all statistical analyses, confirm that the following items are present in the figure legend, table legend, main text, or Methods section.

n/a	Confirmed	
☐	☒	The exact sample size (n) for each experimental group/condition, given as a discrete number and unit of measurement
☐	☒	A statement on whether measurements were taken from distinct samples or whether the same sample was measured repeatedly
☐	☒	The statistical test(s) used AND whether they are one- or two-sided<br>*Only common tests should be described solely by name; describe more complex techniques in the Methods section.*
☐	☒	A description of all covariates tested
☒	☐	A description of any assumptions or corrections, such as tests of normality and adjustment for multiple comparisons
☐	☒	A full description of the statistical parameters including central tendency (e.g. means) or other basic estimates (e.g. regression coefficient) AND variation (e.g. standard deviation) or associated estimates of uncertainty (e.g. confidence intervals)
☐	☒	For null hypothesis testing, the test statistic (e.g. F, t, r) with confidence intervals, effect sizes, degrees of freedom and P value noted<br>*Give P values as exact values whenever suitable.*
☒	☐	For Bayesian analysis, information on the choice of priors and Markov chain Monte Carlo settings
☒	☐	For hierarchical and complex designs, identification of the appropriate level for tests and full reporting of outcomes
☒	☐	Estimates of effect sizes (e.g. Cohen's d, Pearson's r), indicating how they were calculated

Our web collection on statistics for biologists contains articles on many of the points above.

Software and code

Policy information about availability of computer code

Data collection	Flow cytometry data was collected using CytExpert (Beckman Coulter) and FACSDiva (v8, BD Biosciences). FACSAria Fusion (BD Biosciences) was used for cell sorting. 10x Genomics 5' V(D)J Kits were used on the Chromium machine and sequenced using the Illumina MiSeq. Orbitrap Fusion Lumos (Thermo Fisher Scientific) was used for mass spectrometry. BLltzTM system (ForteBio, USA) was used for binding assays. lncucyte ZOOM and S3 (Essence BioScience) was used in T cell cytotoxicity assays. Aperio CS-O slide scanner (Leica Biosystems) was used in scanning IHC slides. Sanger sequencing was performed at CHOP NapCore.
Data analysis	Flow cytometry analysis was performed using FlowJo (v10.7.l, BD Biosciences), R Studio, and Microsoft Excel were used to analyze data. Cellranger VDJ was used to analyze single-cell TCR data. SequestHT algorithm in the Proteome Discoverer (v2.1 and v2.4, ThermoFisher) software was used for LC/MS/MS analysis. NetMHC-4.0 was used in pMHC binding predictions. PH LAT 1.1 was used for HLA typing. Blitz Pro TM software was used to analyze scFv binding data. PyMOL v2.4.1 and RosettaMHC were used for structural modeling. DNA constructs and sequencing data analyzed using SnapGene v5.2 and Benchling. Gene Ontology analyses were performed using PANTHER (v16.0). Crystallization model building and refinement were performed using COOT and Phenix (v1.19.2), respectively. Our algorithms ShinyNAP and sCRAP are described in methods and referenced, and have been made available to reviewers at: https://marisshiny.research.chop.edu/sCRAP/

For manuscripts utilizing custom algorithms or software that are central to the research but not yet described in published literature, software must be made available to editors and reviewers. We strongly encourage code deposition in a community repository (e.g. GitHub). See the Nature Portfolio guidelines for submitting code & software for further information.

Data

Policy information about availability of data

All manuscripts must include a data availability statement. This statement should provide the following information, where applicable:

- Accession codes, unique identifiers, or web links for publicly available datasets
- A description of any restrictions on data availability
- For clinical datasets or third party data, please ensure that the statement adheres to our policy

- Proteomics data is available on PRIDE database as follows:
Project Name: Neuroblastoma HLA class I Immunopeptidomics
Project accession: PXD027182
Project DOI: 10.6019/PXD027182
Reviewer username: reviewer_pxd027182@ebi.ac.uk
Reviewer password: JHiSKklT
All proteins structures are available in the Protein Data Bank under accession codes HLA-A*02:01/LLLPLLPPL (PDB: 7MJ6), HLA-A*02:01/LLPLLPPLSP (PDB: 7MJ7), HLA-A*02:01/LLPLLPPLSPS (PDB: 7MJ8), HLA-A*02:01/LLPRLPPL (PDB: 7MJ9), and HLA-A*24:02/QYNPIRTTF (PDB: 7MJA).

Field-specific reporting

Please select the one below that is the best fit for your research. If you are not sure, read the appropriate sections before making your selection.

☒ Life sciences ☐ Behavioural & social sciences ☐ Ecological, evolutionary & environmental sciences

For a reference copy of the document with all sections, see nature.com/documents/nr-reporting-summary-flat.pdf

Life sciences study design

All studies must disclose on these points even when the disclosure is negative.

Sample size	Sample size for murine studies is based on our review of preclinical trials and publication 10.1158/0008-5472.CAN-16-0122 that show that N=6/arm is an ethically sound and statistically rigorous sample size for preclinical therapeutic trials. Cytotoxicity and cytokine assays were performed using T cells from n=3 independent donors, a sample size that allows for statistically significant differences to be ascertained within the context of donor variability.
Data exclusions	No data were excluded.
Replication	In-vivo studies were conducted twice in independent experiments with similar results. All mass spec samples were run in triplicate with similar conclusions, and all binding assays were performed in triplicate with matching results. All T cell assays were performed using three individual donors undergoing separate lentiviral transductions, and cytotoxicity and cytokine assays were performed with at least three biological and three technical replicates for all reported experiments with similar results between individual donors. Other in-vitro assays were typically reproduced at least three times in independent experiments.
Randomization	Randomization of samples for the repeated studies was performed by selecting tumor sizes within a range of 0.06cm^3-0.19cm^3 for SKNAS and COG-N-564x. Using Excel, the tumors were selected and placed into n=6 groups to generate a median size of 0.11cm^3-0.12cm^3 for COG-N-564x and SKNAS. A range of 0.12cm^3-0.24cm^3 was selected for NBSD to generate a median size of 0.14cm^3-0.15cm^3.
Blinding	During in-vivo studies, blinding was performed during tumor measurement. For other experiments (including in-vitro experiments), blinding could not be completed due to lack of personnel necessary for facilitating adequate blinding. Data analysis was completed with familiarity of experimental conditions and groups.

Reporting for specific materials, systems and methods

We require information from authors about some types of materials, experimental systems and methods used in many studies. Here, indicate whether each material, system or method listed is relevant to your study. If you are not sure if a list item applies to your research, read the appropriate section before selecting a response.

Materials & experimental systems

n/a	Involved in the study
☐	☒ Antibodies
☐	☒ Eukaryotic cell lines
☒	☐ Palaeontology and archaeology
☐	☒ Animals and other organisms
☐	☒ Human research participants
☒	☐ Clinical data
☒	☐ Dual use research of concern

Methods

n/a	Involved in the study
☐	☒ ChIP-seq
☐	☒ Flow cytometry
☒	☐ MRI-based neuroimaging

Antibodies

Antibodies used

The following fluorophore-conjugated antibodies were used ("h" prefix refers to anti-human): hCD3 (Clone F7.2.38, Dako, A0452, 1:100), hPHOX2B (Clone EPR14423, Abcam, ab183741, 1:500), hHLA-ABC (Clone W6/32, Abcam, ab70328, 1:1200), FITC anti-influenza A virus nucleoprotein (Clone D67J Abcam, ab210526, 1:100), APC hCD137 (4-1BB) (Clone 4B4-1, Biolegend, 309809, 1:100), FITC hCD3 (UCHT1, Biolegend, 300405, 1:100), Brilliant Violet 510 hCD19 (HIB19, Biolegend, 302241, 1:100), Brilliant Violet 605 hCD8a (RPA-T8, Biolegend, 301039, 1:100), PE-Cyanine5.5 hCD4 (S3.5, Invitrogen, MHCD0418, 1:100).

Validation

All antibodies used in immunohistochemistry, flow cytometry, and immunofluorescence were titrated and validated for intended use.
Antibodies used for flow cytometry were compared to unstained and isotype controls, when applicable.
Manufacturer validation statements can be found below for the corresponding antibodies.
hCD3: https://www.agilent.com/en/product/immunohistochemistry/antibodies-controls/primary-antibodies/cd3-(concentrate)-76649
hPHOX2B: https://www.abcam.com/phox2b-antibody-epr14423-c-terminal-ab183741.html
hHLA-ABC: https://www.abcam.com/hla-class-1-abc-antibody-emr8-5-ab70328.html
FITC anti-influenza A virus nucleoprotein: https://www.abcam.com/fitc-influenza-a-virus-nucleoprotein-antibody-d67j-ab210526.html
APC hCD137: https://www.biolegend.com/en-us/products/apc-anti-human-cd137-4-1bb-antibody-3910?GroupID=BLG2623
FITC hCD3: https://www.biolegend.com/en-us/products/fitc-anti-human-cd3-antibody-863
Brilliant Violet 510 hCD19: https://www.biolegend.com/en-us/products/brilliant-violet-510-anti-human-cd19-antibody-8004?GroupID=BLG5913
Brilliant Violet 605 hCD8a: https://www.biolegend.com/en-ie/products/brilliant-violet-605-anti-human-cd8a-antibody-7651
PE-Cyanine5.5 hCD4: https://www.thermofisher.com/antibody/product/CD4-Antibody-clone-S3-5-Monoclonal/MHCD0418

Eukaryotic cell lines

Policy information about cell lines

Cell line source(s)

SK-N-AS, SK-N-FI, and NB-SD neuroblastoma cancer cell lines were obtained from the Maris Lab cell line bank. Other human cancer cell lines, including 293T (human embryonic kidney), Jurkat (acute T cell leukemia), SW620 (Dukes' type C, colorectal adenocarcinoma), HEPG2 (hepatocellular carcinoma), and KATO III (gastric carcinoma) were obtained from American Type Culture Collection (ATCC). Platinum-A (Plat-A) cells were obtained from Cell Biolabs. Primary human T cells were obtained from anonymous donors through the Human Immunology Core at the Perelman School of Medicine at the University of Pennsylvania (Philadelphia, Pennsylvania).

Authentication

Short-tandem repeat profiling of cell lines was provided by ATCC upon purchase, and Maris Lab cell line bank lines were regularly authenticated by short-tandem repeat profiling.

Mycoplasma contamination

Cells were regularly tested for mycoplasma contamination and found to be negative.

Commonly misidentified lines
(See ICLAC register)

No commonly misidentified cell lines were used to the authors' knowledge.

Animals and other organisms

Policy information about studies involving animals; ARRIVE guidelines recommended for reporting animal research

Laboratory animals

NSG (NOD SCIO Gamma) 6-8 week old Females, Jackson Labs Stock number 005557. Mice were kept in a 12 hour light/dark cycle at 20-23°C with 30-70% humidity.

Wild animals

No wild animals were used in this study.

Field-collected samples

No field-collected samples were used in this study.

Ethics oversight

All experiments were conducted under a protocol approved by the Children’s Hospital of Philadelphia Institutional Review Board, and all in-vivo experiments were conducted under conditions approved by the Institutional Animal Care and Use Committee at the Children's Hospital of Philadelphia.

Note that full information on the approval of the study protocol must also be provided in the manuscript.

Human research participants

Policy information about studies involving human research participants

Population characteristics	Patient-derived xenografts and high-risk neuroblastoma tumors used for immunopeptidomics and/or in-vivo models were obtained through the ongoing Children's Oncology Group (COG; https://childrensoncologygroup.org/) neuroblastoma biobanking study NCT00904241. All newly diagnosed patients with suspected neuroblastoma, suspected ganglioneuroblastoma, or suspected ganglioneuroma/maturing subtype seen at COG institutions are eligible for this study. Total human primary T cells (CD4/8+) were obtained from the Human Immunology Core at the Perelman School of Medicine at the University of Pennsylvania.
Recruitment	Recruitment for COG biobanking study NCT00904241 was conducted according to study design as indicated on the ClinicalTrials.gov homepage: https://clinicaltrials.gov/show/NCT00904241
Ethics oversight	For human tissue samples, informed consent from each research subject or legal guardian was obtained for each deidentified tumor and blood sample used in this study through the COG neuroblastoma biobanking study NCT00904241

Note that full information on the approval of the study protocol must also be provided in the manuscript.

ChIP-seq

Data deposition

☒ Confirm that both raw and final processed data have been deposited in a public database such as GEO.

☐ Confirm that you have deposited or provided access to graph files (e.g. BED files) for the called peaks.

Data access links<br>*May remain private before publication.*	*For "Initial submission" or "Revised version" documents, provide reviewer access links. For your "Final submission" document, provide a link to the deposited data.*
Files in database submission	*Provide a list of all files available in the database submission.*
Genome browser session<br>(e.g. UCSC)	*Provide a link to an anonymized genome browser session for "Initial submission" and "Revised version" documents only, to enable peer review. Write "no longer applicable" for "Final submission" documents.*

Methodology

Replicates	*Describe the experimental replicates, specifying number, type and replicate agreement.*
Sequencing depth	*Describe the sequencing depth for each experiment, providing the total number of reads, uniquely mapped reads, length of reads and whether they were paired- or single-end.*
Antibodies	*Describe the antibodies used for the ChIP-seq experiments; as applicable, provide supplier name, catalog number, clone name, and lot number.*
Peak calling parameters	*Specify the command line program and parameters used for read mapping and peak calling, including the ChIP, control and index files used.*
Data quality	*Describe the methods used to ensure data quality in full detail, including how many peaks are at FDR 5% and above 5-fold enrichment.*
Software	*Describe the software used to collect and analyze the ChIP-seq data. For custom code that has been deposited into a community repository, provide accession details.*

Flow Cytometry

Plots

Confirm that:

☒ The axis labels state the marker and fluorochrome used (e.g. CD4-FITC).

☒ The axis scales are clearly visible. Include numbers along axes only for bottom left plot of group (a 'group' is an analysis of identical markers).

☒ All plots are contour plots with outliers or pseudocolor plots.

☒ A numerical value for number of cells or percentage (with statistics) is provided.

Methodology

Sample preparation	Cells were harvested and washed once with PBS before resuspension in FACS buffer with appropriate staining reagents or multimers. After 30 minutes of incubation at 4° C, cells were washed three times with PBS before resuspension in FACS

	buffer with 1% formalin fixation. For samples with quantified cell numbers, counting beads were added (CountBright Absolute Counting Beads (Invitrogen, C36950, Lot: 2207530) before sample analysis. Cell viability was determined using LIVE/DEAD Violet Dead Cell Stain (Invitrogen, L34955, Lot: 2179253, 1:1000).
Instrument	Beckman Coulter CytoFLEX S (C01161), Beckman Coulter CytoFLEX LX (C40324), BD Biosciences LSR II, Applied Biosystems Attune Acoustic Focusing Cytometer, BD Biosciences FACSJazz Cell Sorter.
Software	Collection was performed using CytExpert (Beckman Coulter) and FACSDiva (v8, BD Biosciences). Flow cytometry analysis was performed using FlowJo v10.7.1 (BD Biosciences).
Cell population abundance	All sorted populations were often stained and re-analyzed using flow cytometry to ensure phenotypic purity.
Gating strategy	All samples were first gated on FSC/SSC lymphocyte populations, single cells (Using SSC-H/SSC-H), and then live cells (negative V450 channel, LIVE/DEAD Violet Dead Cell Stain, Invitrogen). For neuroblastoma cells infected by H1N5 virus, live, single cells were gated by positive staining for FITC virus nucleoprotein (NP) antibody. For TCRs, live, lymphocyte singlets were gated to identify antigen-specific T cells. For transduced CAR T cells, live, single lymphocytes cells were gated using tagged MHC multimers to identify cells transduced with antigen-specific receptors and to determine cross-reactivity.

☒ Tick this box to confirm that a figure exemplifying the gating strategy is provided in the Supplementary Information.
